# Protein Lactylation in Cancer: From Mechanisms to Therapy

**DOI:** 10.1002/mco2.71106

**Published:** 2026-10-31

**Authors:** Wentian Huang, Ping Hu, Shun Wang, Xide Zhang, Ziran Ye, Shengqiang Chen, Fu Gao, Yuanyuan Chen, Yanyong Yang

**Affiliations:** ^1^ Department of Radiation Medicine Faculty of Naval Medicine Naval Medical University Shanghai China; ^2^ School of Public Health China Medical University Shenyang China; ^3^ Department of Hepatic Surgery VI Eastern Hepatobiliary Surgery Hospital Naval Medical University Shanghai China; ^4^ Shanghai Key Laboratory of Nautical Medicine and Translation of Drugs and Medical Devices Shanghai China

**Keywords:** DNA damage response, lactate metabolism, protein lactylation, therapeutic resistance, tumor microenvironment

## Abstract

Protein lactylation has emerged as a metabolically responsive posttranslational modification linking lactate accumulation to epigenetic and functional adaptation in cancer. Initially identified on histones, lactylation is now recognized on diverse nonhistone proteins involved in DNA damage repair, immune regulation, redox homeostasis, metabolic remodeling, and cellular plasticity. However, current evidence remains fragmented across treatment modalities, and many reported lactylation events lack site‐specific functional validation and translational assessment. This review addresses this gap by integrating the metabolic and enzymatic basis of protein lactylation with its roles in chemotherapy, radiotherapy, targeted therapy, and immunotherapy. We critically examine how lactylation may promote tumor persistence under therapeutic pressure by enhancing genome maintenance, immune evasion, stress‐survival signaling, metabolic adaptability, and tumor microenvironment remodeling. We also evaluate emerging strategies targeting lactate production and transport, lactylation‐regulating enzymes, and functionally validated substrates, including their potential use in combination therapies. Finally, we discuss major barriers to clinical translation, including incomplete characterization of regulatory enzymes, limitations in site‐specific lactylome mapping, insufficient causal validation, lack of clinically qualified biomarkers, tumor metabolic heterogeneity, and challenges in achieving therapeutic selectivity. Collectively, protein lactylation represents an emerging mechanistic framework for understanding cancer treatment adaptation and a potentially targetable axis whose clinical relevance requires rigorous validation.

## Graphical abstract

Increased glycolysis and lactate accumulation promote histone and nonhistone protein lactylation, linking tumor metabolic reprogramming to treatment adaptation. Lactylation enhances DNA repair, suppresses antitumor immunity, strengthens antioxidant defenses, and facilitates cell‐cycle checkpoint escape, collectively promoting resistance to chemotherapy, radiotherapy, targeted therapy, and immunotherapy.

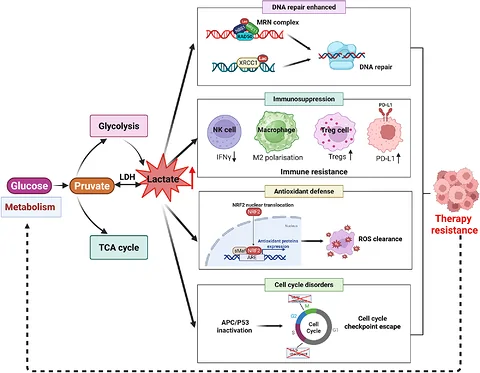

## 1 Introduction

Otto Warburg's description of aerobic glycolysis established a lasting framework for understanding tumor metabolism [1]. For decades, lactate was regarded primarily as a metabolic by‐product [2]. This view has undergone substantial revision. Lactate is now recognized as a signaling metabolite. It influences chromatin regulation, immune behavior, and stress adaptation in both cancer cells and the tumor microenvironment (TME) [3, 4, 5]. The identification of histone lysine lactylation (Kla) marked a pivotal advance [6], establishing a mechanistic link between metabolic reprogramming and epigenetic control and opening new avenues for investigating how tumors convert metabolic pressure into stable regulatory outputs.

Protein lactylation is neither a rare chemical by‐product nor a histone‐restricted event [7, 8, 9]. It represents a regulated posttranslational modification shaped by metabolic context, enzymatic activity, and substrate accessibility [9, 10, 11, 12, 13]. p300/CBP remains the best‐characterized writer of lysine lactylation [6, 13, 14, 15]. Class I HDACs and selected sirtuins function as erasers under specific conditions [10]. Additional enzyme systems contribute to context‐specific lactylation events [12, 16, 17, 18, 19, 20, 21]. Proteomic studies have expanded the known lactylome beyond histones to include nonhistone proteins involved in metabolism, DNA damage response (DDR), transcriptional regulation, and immune signaling [7, 8, 13, 15, 22]. This expanded substrate repertoire suggests that lactylation modulates tumor behavior at multiple regulatory layers.

The functional significance of protein lactylation is particularly evident in the TME [4, 8, 23, 24]. Hypoxia, nutrient limitation, acidosis, and immune competition converge in this environment. Protein lactylation functions as a molecular interface under these conditions. It links lactate‐rich metabolic states to adaptive cellular outputs [8, 25, 26]. Histone lactylation has been associated with transcriptional programs governing immune suppression, angiogenesis, stemness, and stress tolerance [27, 28, 29, 30, 31]. Nonhistone lactylation can modulate enzymatic activity, protein turnover, subcellular localization, and protein–protein interactions [32, 33, 34]. These findings indicate that protein lactylation extends beyond a passive marker of elevated lactate. It constitutes a functional regulatory layer through which metabolic stress is translated into phenotypic plasticity and adaptive tumor behavior.

The clinical relevance of this framework becomes especially apparent in therapeutic settings [35, 36]. In selected tumor contexts, anticancer therapies can reshape tumor metabolism in ways that increase lactate availability and favor lactylation‐dependent survival mechanisms [6, 37]. These changes influence lactate production, transport, and downstream protein modification [6, 16]. Chemotherapy and ionizing radiation may enhance glycolytic stress and lactate accumulation [35, 38, 39]. They simultaneously induce DNA damage, creating conditions permissive for lactylation‐dependent repair and survival pathways [40, 41, 42]. Targeted therapies can be compromised by metabolic bypass programs that preserve lactate flux [43, 44, 45]. In the context of immunotherapy, lactate‐rich TMEs suppress cytotoxic T‐cell function and promote regulatory T‐cell expansion [46, 47, 48, 49, 50]. Protein lactylation of immune checkpoint proteins and transcription factors may contribute to these effects [27, 51, 52, 53, 54]. These observations suggest that protein lactylation is not merely a consequence of tumor metabolism but an active participant in treatment adaptation and resistance (Figure 1).

**FIGURE 1:**
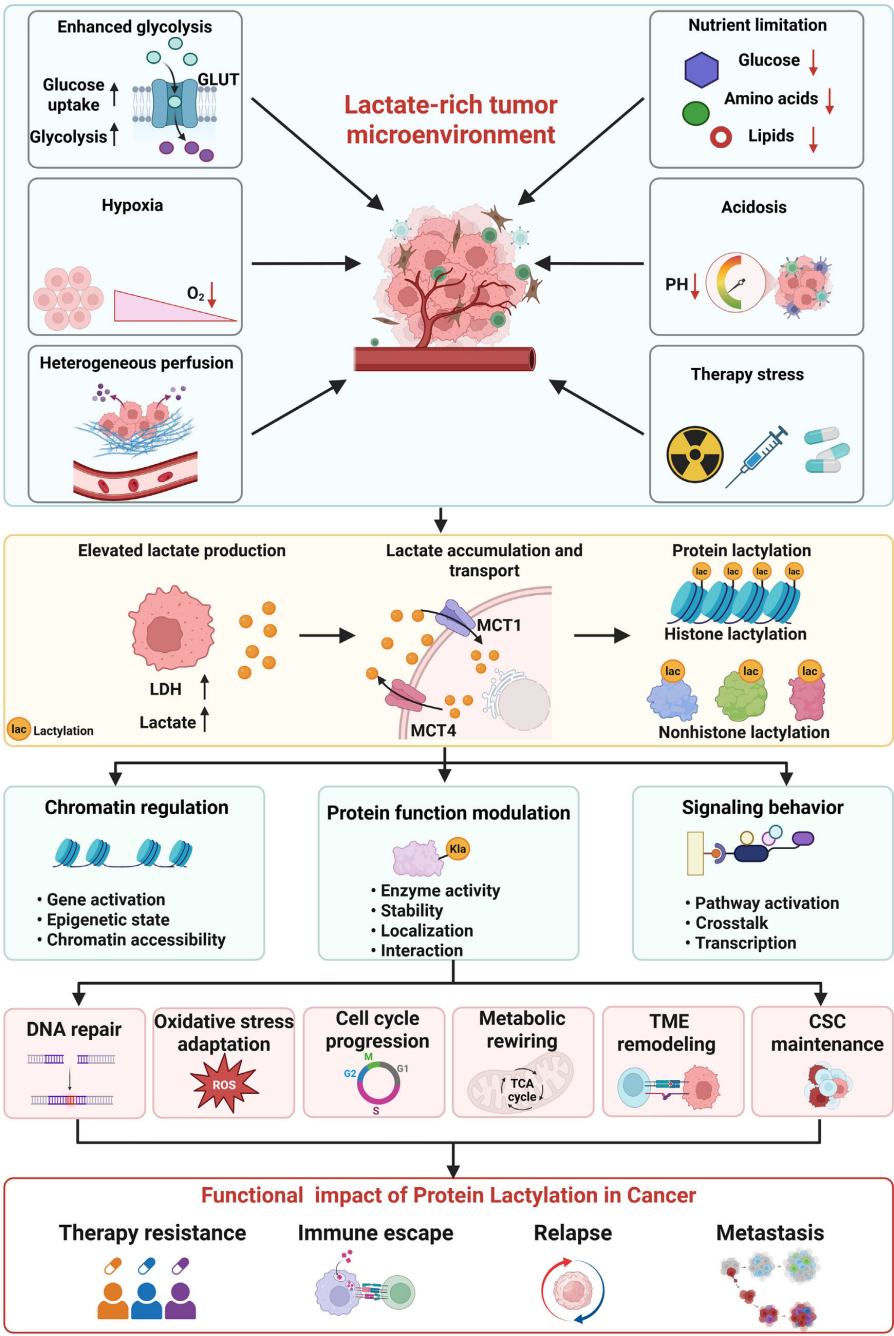
Protein lactylation as a metabolic–epigenetic interface in cancer. In lactate‐rich tumors, multiple converging microenvironmental pressures—including enhanced aerobic glycolysis driven by GLUT‐mediated glucose uptake, hypoxia, heterogeneous perfusion, nutrient depletion, extracellular acidosis, and therapy‐induced metabolic stress—cooperate to establish a lactate‐rich tumor microenvironment. The resulting intracellular lactate, generated via lactate dehydrogenase (LDH)‐mediated reduction of pyruvate and imported or exported through monocarboxylate transporters MCT1 and MCT4, provides the substrate for site‐specific lysine lactylation (Kla) on both histone and nonhistone proteins. Histone lactylation reprograms chromatin accessibility and gene activation, whereas nonhistone lactylation modulates enzyme activity, protein stability, subcellular localization, and protein–protein interactions, collectively reshaping oncogenic signaling and transcriptional output. These molecular events converge on six core cellular processes—DNA damage repair, oxidative stress adaptation, cell cycle progression, metabolic rewiring, tumor microenvironment (TME) remodeling, and cancer stem cell (CSC) maintenance—several of which further reinforce glycolytic flux and lactate production through positive feedback. The integrated functional output of this regulatory architecture manifests as four clinically relevant phenotypes: therapy resistance, immune escape, tumor relapse, and metastasis. Abbreviations: GLUT, glucose transporter; TCA, tricarboxylic acid cycle.

Against this background, the present review examines protein lactylation as a dynamic regulator of therapeutic response and resistance in cancer. Unlike prior reviews focused on tumor progression, we organize the evidence through a treatment‐centric lens, systematically addressing how lactylation is engaged across chemotherapy, radiotherapy, targeted therapy, and immunotherapy (Sections 2 and 3); the shared resistance mechanisms it enables (Section 4); current and emerging targeting strategies (Section 5); and key barriers to clinical translation (Section 6). By integrating recent evidence within this framework, we aim to provide a mechanistic foundation for understanding the metabolism–epigenetics–therapeutic response interface and to guide the rational development of lactylation‐targeted cancer therapies.

## 2 Molecular Basis of Protein Lactylation and Its Relevance to Malignant Phenotypes

In solid tumors, chronic hypoxia, acidosis, nutrient limitation, and uneven perfusion impose sustained metabolic stress on both malignant and stromal cells [55, 56, 57]. Under these conditions, tumor progression depends not only on altered metabolite production, but also on mechanisms that convert metabolic imbalance into durable regulatory outputs. Protein lactylation has emerged as one such mechanism. Rather than serving as a passive consequence of lactate accumulation, lactylation is increasingly recognized as a metabolically responsive posttranslational modification that influences chromatin state, protein function, and cellular adaptation [58, 59]. In several preclinical settings, increased lactate availability under hypoxia, inflammatory stimulation, or therapy‐related stress has been associated with elevated histone lactylation, including H3K18la, together with induction of genes linked to immune modulation, stress adaptation, and malignant plasticity [60, 61, 62, 63, 64]. These observations support the concept that lactylation participates in how tumors integrate metabolic stress with microenvironmental responses, though the functional importance of individual lactylation events requires site‐specific validation.

### 2.1 Chemical Basis and Stereochemical Diversity of Protein Lactylation

#### 2.1.1 Lactyl‐Group Donors and Chemical Properties

Protein lactylation involves covalent addition of a lactyl group (–CO–CH(OH)–CH_3_) to the ε‐amino group of lysine residues. This modification requires an activated lactyl donor, most commonly lactyl‐CoA [6, 9, 20, 65]. The biosynthesis of lactyl‐CoA represents a critical metabolic checkpoint linking glycolytic flux to protein modification. Two lactyl‐CoA synthetases have been identified: acyl‐CoA synthetase short‐chain family member 2 (ACSS2) and GTP‐specific succinyl‐CoA synthetase (GTPSCS). ACSS2 functions as a lactyl‐CoA synthetase, converting lactate into lactyl‐CoA through a two‐step reaction [19]. These observations provide a biochemical link between glycolytic lactate production and enzymatic protein lactylation, although the relative contribution of individual lactyl‐CoA‐generating enzymes is likely to depend on cell type and metabolic context.

Compared with acetylation, lactylation introduces a larger mass shift (+72 vs. +42 Da) and an additional hydroxyl group [66, 67]. These structural features may alter lysine charge neutralization, local protein conformation, and protein–protein or protein–chromatin interactions in a site‐dependent manner [32, 67, 68, 69]. Lactylation and acetylation at the same or adjacent lysine residues may therefore generate distinct regulatory outcomes, although these effects require site‐specific functional validation.

#### 2.1.2 Stereochemical Forms and Dnalytical Distinction

Lysine lactylation comprises stereochemically distinct forms with different biochemical origins. l‐Lactyllysine (K_L‐la_) is principally associated with glycolysis‐derived l‐lactate and enzymatic lactyl‐group transfer [9, 70, 71]. By contrast, d‐lactyllysine (K_D‐la_) is generated mainly through a nonenzymatic pathway involving lactoylglutathione (LGSH), a reactive intermediate of the glyoxalase system. Methylglyoxal (MG) can also directly modify lysine through Michael addition to form N‐ε‐carboxyethyllysine (Kce) However, Kce is a structurally related lysine adduct rather than a stereoisomer of lactyllysine and should be distinguished from K_L‐la_ and K_D‐la_ in biochemical and analytical studies [10].

This stereochemical distinction has implications for interpreting cancer‐associated lactylation [72, 73]. Changes detected by pan‐lactyllysine antibodies or nonstereospecific proteomic workflows may not establish which lactyllysine form is altered or which upstream metabolic route is responsible. The enzymatic regulation and stress‐dependent generation of these modifications are discussed in Section 2.2, whereas their functional consequences for transcription, metabolism, proteostasis, and stress‐response signaling are considered in Section 2.3.

### 2.2 Regulatory Machinery of Protein Lactylation

Tumor cells use a multilayered lactylation‐regulatory system to translate transient metabolic changes into sustained biological effects. Current evidence supports a model that includes enzymatic installation and removal of lactyl marks, reader‐mediated recognition, and reactive metabolite‐driven lactyl‐group transfer under stress conditions [71] (Figure 2A).

**FIGURE 2:**
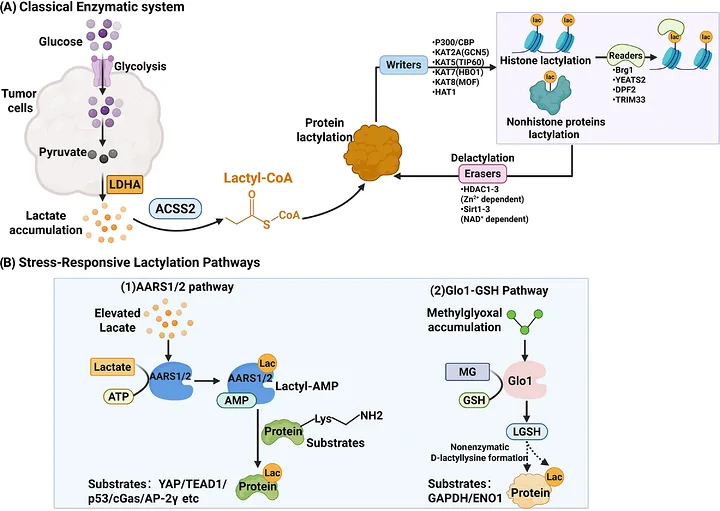
Metabolic and enzymatic basis of protein lactylation in cancer. Enzymatic and nonenzymatic mechanisms of protein lactylation. (A) In the canonical lactyl‐CoA‐dependent pathway, glycolysis‐derived lactate is converted by ACSS2 into lactyl‐CoA, which serves as the acyl donor for a family of lysine acetyltransferase writers—including p300/CBP, KAT2A, KAT5, KAT7, and KAT8—to deposit lactyl marks on histone and nonhistone substrates. Installed marks are recognized by reader proteins (Brg1, YEATS2, DPF2, TRIM33) that couple lactylation to chromatin remodeling and transcriptional activation, while Zn^2^^+^‐dependent Class I HDACs and NAD^+^‐dependent sirtuins (SIRT1–3) dynamically reverse the modification, rendering lactylation responsive to the metabolic state of the cell. (B) Under acute metabolic stress, two lactyl‐CoA‐independent routes expand the lactylation landscape. Alanyl‐tRNA synthetases AARS1/2 directly activate lactate to a lactyl‐AMP intermediate and transfer the lactyl group to nuclear substrates such as YAP and p53, bypassing the need for CoA conjugation. In parallel, methylglyoxal accumulation drives Glo1‐mediated formation of lactoylglutathione (LGSH), which nonenzymatically donates a d‐lactyl group to abundant metabolic proteins. Together, these complementary pathways establish protein lactylation as a multi‐input metabolic sensor whose substrate range and stereo‐chemical output are determined by the specific upstream metabolic context. Abbreviations: GAPDH, glyceraldehyde‐3‐phosphate dehydrogenase; YEATS2, YEATS domain‐containing protein 2.

#### 2.2.1 Enzymatic “Writer–Eraser–Reader” System

##### 2.2.1.1 Writers

Lactylation writers can be classified into two major categories based on their catalytic mechanisms: lysine acyltransferases (KATs) that utilize lactyl‐CoA, and alanyl‐tRNA synthetases (AARS1/2) that directly employ lactate and ATP [11, 12]. Among lactyl‐CoA‐dependent writers, the histone acetyltransferase p300/CBP remains the best‐validated lactylation writer [15]. It exhibits broad acyl‐donor promiscuity and concentration‐dependent use of lactyl‐CoA [74]. Other KATs spanning multiple families—including the GNAT family (KAT2A/GCN5), the MYST family (KAT5/TIP60, KAT7/HBO1, KAT8/MOF), and HAT1—support lactylation in selected contexts [11, 12, 39, 75, 76]. KAT2A forms a functional complex with ACSS2, creating localized lactyl‐CoA microenvironments [19].

##### 2.2.1.2 Erasers

The removal of lactylation marks is executed by two enzyme families with distinct cofactor requirements and stereochemical preferences. Class I HDACs (HDAC1–3) exhibit Zn^2^^+^‐dependent delactylase activity with >3.5‐fold higher catalytic efficiency toward K_D‐la_ compared with K_L‐la_ [77, 78], whereas SIRT1–3 function as NAD^+^‐dependent delactylases with a preference for K_L‐la_ [72, 79, 80, 81]. These differences suggest that the persistence of individual lactyllysine forms depends on both their route of formation and the local availability and activity of specific delactylases. Because hypoxia and metabolic stress alter cellular redox state and NAD^+^ availability, delactylase activity may vary across tumor compartments and treatment conditions [61, 82, 83, 84]. Recent studies indicate that HDACs can exhibit lactate‐dependent bifunctionality: while functioning as delactylases in vitro, HDAC1–3 may shift to lactyltransferase activity at physiological lactate concentrations (>0.5 mM) [10, 77]. However, the net intracellular activities of these enzymes cannot be inferred solely from in vitro substrate preferences.

##### 2.2.1.3 Readers

Reader proteins connect lactylation marks with downstream functional responses. a core ATPase subunit of the SWI/SNF chromatin‐remodeling complex, recognizes H3K18la through its bromodomain, linking this histone mark to chromatin remodeling and transcriptional regulation [67]. DPF2 employs its plant homeodomain (PHD) finger domain to anchor H3K14la within a hydrophobic pocket, promoting oncogene expression in cervical cancer [68]. TRIM33 utilizes its bromodomain with a critical glutamate residue (E981) for hydrogen‐bonding recognition of H3K18la, thereby regulating macrophage polarization [69]. The full spectrum of lactylation readers, particularly those recognizing nonhistone substrates, remains to be systematically defined.

#### 2.2.2 Stress‐Responsive and Nonenzymatic Pathways

In addition to lactyl‐CoA‐dependent KAT activity, protein lactylation can arise through alternative enzymatic and nonenzymatic routes whose relative contributions vary with lactate availability, glycolytic flux, and carbonyl stress [9, 70] (Figure 2B).

##### 2.2.2.1 AARS1/2 Pathway

Alanyl‐tRNA synthetases (AARS1/2) can accommodate lactate in their substrate‐binding pockets under elevated lactate conditions [17]. They activate lactate as lactyl‐adenosine monophosphate (lactyl‐AMP) and directly transfer lactyl groups to lysine residues, bypassing ACSS2‐ and CoA‐dependent steps [18, 19, 85]. Elevated lactate binding induces a conformational shift that exposes a C‐terminal nuclear localization signal (C‐terminal NLS), promoting nuclear import of AARS1/2 [86]. Once in the nucleus, AARS1/2 can lactylate nuclear proteins such as YAP and TEAD1, activating downstream oncogenic signaling [85]. This pathway allows rapid lactylation responses under acute metabolic stress.

##### 2.2.2.2 Glo1–LGSH Nonenzymatic Pathway

When MG accumulates, glyoxalase 1 (Glo1) catalyzes the reaction between glutathione (GSH) and MG to generate LGSH [87, 88]. LGSH can act as a reactive lactoyl donor, nonenzymatically modifying nearby lysine residues to generate K_D‐la_ [9, 70, 71]. This process can be amplified by cysteine‐mediated relay mechanisms. The Glo1–LGSH route preferentially affects abundant metabolic proteins and represents a stress‐linked chemical mechanism responding to carbonyl stress.

Collectively, these pathways indicate that protein lactylation is a context‐dependent metabolic regulatory process rather than a uniform consequence of lactate accumulation. Lactyl‐CoA‐dependent, AARS1/2‐mediated, and LGSH‐associated pathways may contribute differentially according to tumor type, cellular state, and therapeutic stress, while total lactylation levels alone cannot define the underlying pathway, stereoisomer, or functional consequence. This principle applies across chemotherapy, radiotherapy, targeted therapy, and immunotherapy. In radiotherapy, although irradiation can alter glycolytic and redox states associated with K_L‐la_ and K_D‐la_ formation, their distinct contributions to DDRs or radioresistance remain unclear. Further stereoisomer‐resolved and site‐specific studies are therefore needed.

### 2.3 Functional Impact of Protein Lactylation in Cancer Biology

The chemical routes described above define how lactyl marks are generated, removed, and recognized, but do not by themselves establish biological function. The functional significance of protein lactylation in cancer is better understood through the regulatory outputs produced by specific, site‐resolved modifications. Current evidence implicates lactylation in transcriptional reprogramming, metabolic adaptation, proteostasis, oncogenic and stress‐response signaling, DDRs, and immune regulation. These categories provide a framework for evaluating how lactate‐rich or treatment‐stressed tumor cells acquire plasticity and therapy tolerance, while distinguishing mechanistically validated events from broader lactylome associations.

#### 2.3.1 Transcriptional Reprogramming

Among lactylated substrates, histones are comparatively well characterized with respect to chromatin regulation and gene transcription. Histone lactylation has been associated with increased chromatin accessibility at selected loci and context‐dependent transcriptional activation [89, 90, 91, 92]. Available evidence suggests that these effects may involve reader‐mediated recruitment of chromatin‐remodeling machinery, although the relevant readers, genomic contexts, and causal consequences remain incompletely defined.

Histone lactylation is not uniformly distributed across the genome but exhibits site‐ and locus‐specific enrichment. In hepatocellular carcinoma (HCC), lactate accumulation increases H3K18la enrichment at promoters of PD‐L1, VEGFA, and ARG1, linking lactate metabolism to immune evasion and angiogenesis [93, 94, 95, 96]. Mechanistically, H3K18la recruits the chromatin remodeler Brg1, which promotes chromatin accessibility and transcriptional activation [67, 97]. In triple‐negative breast cancer (TNBC), H4K12la associates with the SLFN5 promoter, suppressing SLFN5 expression and promoting tumor cell proliferation [98, 99, 100]. In liver cancer stem cells, H3K56la correlates with stemness‐associated factors such as OCT4 and NANOG [30, 101].

Beyond direct transcriptional activation, histone lactylation can regulate posttranscriptional processes. In non‐small cell lung cancer (NSCLC), H3K18la enrichment at the YTHDF2 promoter upregulates YTHDF2, which recognizes m^6^A‐modified SFRP2 transcripts, enhancing glycolysis and tumor stemness [102, 103]. These examples indicate that histone lactylation redirects transcriptional programs in a context‐dependent manner, influencing selected loci involved in immune regulation, angiogenesis, stemness, and stress adaptation. However, the direction and magnitude of these effects depend on histone site, genomic locus, reader recruitment, and tumor type.

#### 2.3.2 Metabolic Adaptation

While histone lactylation regulates transcriptional programs, nonhistone lactylation directly modulates metabolic enzyme activity. Proteomic surveys have identified lysine lactylation on metabolic enzymes, RNA‐binding proteins, cytoskeletal regulators, and DNA repair factors, indicating that lactylation's influence extends beyond chromatin regulation [8, 21, 39, 104, 105].

Glycolysis‐associated proteins such as PKM2, ALDOA, and LDHA are lactylated in cancer systems [106, 107, 108, 109]. In lung adenocarcinoma, LDHA undergoes lysine lactylation at K81 and K318, which enhances its enzymatic activity and promotes lactate production. The increased lactate further elevates global cellular lactylation, thereby establishing a “lactate production–lactylation” positive feedback loop [109]. This modification reinforces glycolytic dependence and supports rapid ATP generation. Conversely, ALDOA–K147la reduces ALDOA enzymatic activity and suppresses glycolytic flux, demonstrating that lactylation does not uniformly enhance metabolism [32, 107, 109]. In TNBC, high lactate dehydrogenase C4 (LDHC4) expression promotes ACAA2–K214la, increasing free fatty acid accumulation and linking lactate‐rich states to lipid metabolism [110]. These findings indicate that lactylation can reshape enzyme behavior in a context‐dependent manner, affecting enzyme localization, protein–protein interactions, or pathway coupling. The metabolic consequence depends on the modified enzyme's position in the pathway, requiring site‐resolved functional studies to distinguish causal regulation from broader lactylome associations.

#### 2.3.3 Proteostasis and Protein Stability Control

A growing body of evidence indicates that lactylation can regulate protein stability by interfering with ubiquitination, E3 ligase recognition, or proteasomal degradation [33, 54]. When lactylation occurs at key lysine residues, it may compete with ubiquitination or alter the binding interface between a substrate and its degradation machinery. In some cases, this stabilizes oncogenic or stress‐response proteins. In other contexts, lactylation may promote noncanonical degradation routes.

##### 2.3.3.1 Stabilization Mechanisms

Protein lactylation stabilizes HIF‐1α by inhibiting ubiquitin‐mediated degradation, allowing continuous activation of angiogenesis‐related genes such as VEGF under hypoxia [22, 54, 111]. Similarly, TFEB lactylation prolongs its half‐life, enhancing autophagy and lysosomal biogenesis [33]. In cervical cancer, DCBLD1–K172la stabilizes DCBLD1 expression, inhibiting autophagic degradation of G6PD and activating the pentose phosphate pathway (PPP) [112].

##### 2.3.3.2 Degradation Mechanisms

Conversely, cGAS lactylation promotes its degradation through a ubiquitin‐independent mechanism, suppressing innate immune sensing [34]. In gastric cancer (GC), METTL16–K229la enhances m^6^A modification of FDX1 mRNA, stabilizing it and inducing cuproptosis, demonstrating an anticancer effect [113].

These cases show that protein lactylation can either prolong or reduce protein half‐life depending on substrate and cellular context. Such proteostasis regulation is particularly relevant because many proteins involved in hypoxia adaptation, immune sensing, and survival signaling are controlled by rapid turnover.

#### 2.3.4 Integration With Oncogenic and Stress‐Response Pathways

Lactylation intersects with major oncogenic and stress‐response pathways, functioning as a molecular bridge between metabolic state and signaling behavior. Rather than operating as an isolated metabolic mark, lactylation reinforces existing signaling circuits by modifying pathway components or their regulators. Examples include HIF‐1α‐related hypoxic adaptation, RHOA‐mediated cytoskeletal remodeling, cGAS‐linked innate immune sensing, and NBS1/MRE11‐associated DDR [13, 34, 39, 54, 114]. These examples suggest that lactylation serves as a molecular interface linking lactate‐rich metabolic states to adaptive signaling outputs. In tumor contexts, this interface supports invasion, immune escape, and survival under hostile conditions. The therapeutic implications of these signaling events, particularly in the context of chemotherapy, radiotherapy, and immunotherapy, are examined in subsequent sections.

The functional consequences of protein lactylation in cancer arise from its ability to integrate metabolic state with transcriptional, metabolic, proteostatic, and signaling outputs. While histone lactylation primarily regulates gene expression through chromatin remodeling, nonhistone lactylation directly modulates enzyme activity, protein stability, and pathway crosstalk. The current evidence base is heterogeneous: histone lactylation marks such as H3K18la are supported by mechanistic studies with reader identification and functional validation, whereas many nonhistone lactylation events remain at the proteomic discovery stage, requiring site‐resolved biochemical characterization and in vivo validation. Understanding the hierarchy of lactylation events—from causal regulators to bystander marks—is critical for prioritizing therapeutic targets [98, 100, 113, 114, 115]. The following sections examine how these lactylation‐driven mechanisms contribute to therapy resistance, immune evasion, and explore strategies for targeting lactylation in cancer treatment.

## 3 Protein Lactylation Dynamics in Cancer Therapy

Anticancer therapies reshape tumor biology dynamically. They modulate oxygen availability, redox balance, nutrient supply, perfusion, and stromal–immune interactions. These changes collectively influence lactate production, transport, and subsequent protein lactylation. Protein lactylation may facilitate tumor survival during treatment by enhancing DDR, immune evasion, metabolic adaptability, and stress‐survival signaling. Building upon the functional framework in Section 2, this section explores how the lactate–lactylation axis engages across major treatment modalities and how specific lactylation events contribute to tumor persistence, compensatory network reconfiguration, and acquired resistance under therapeutic pressure (Figure 3).

**FIGURE 3:**
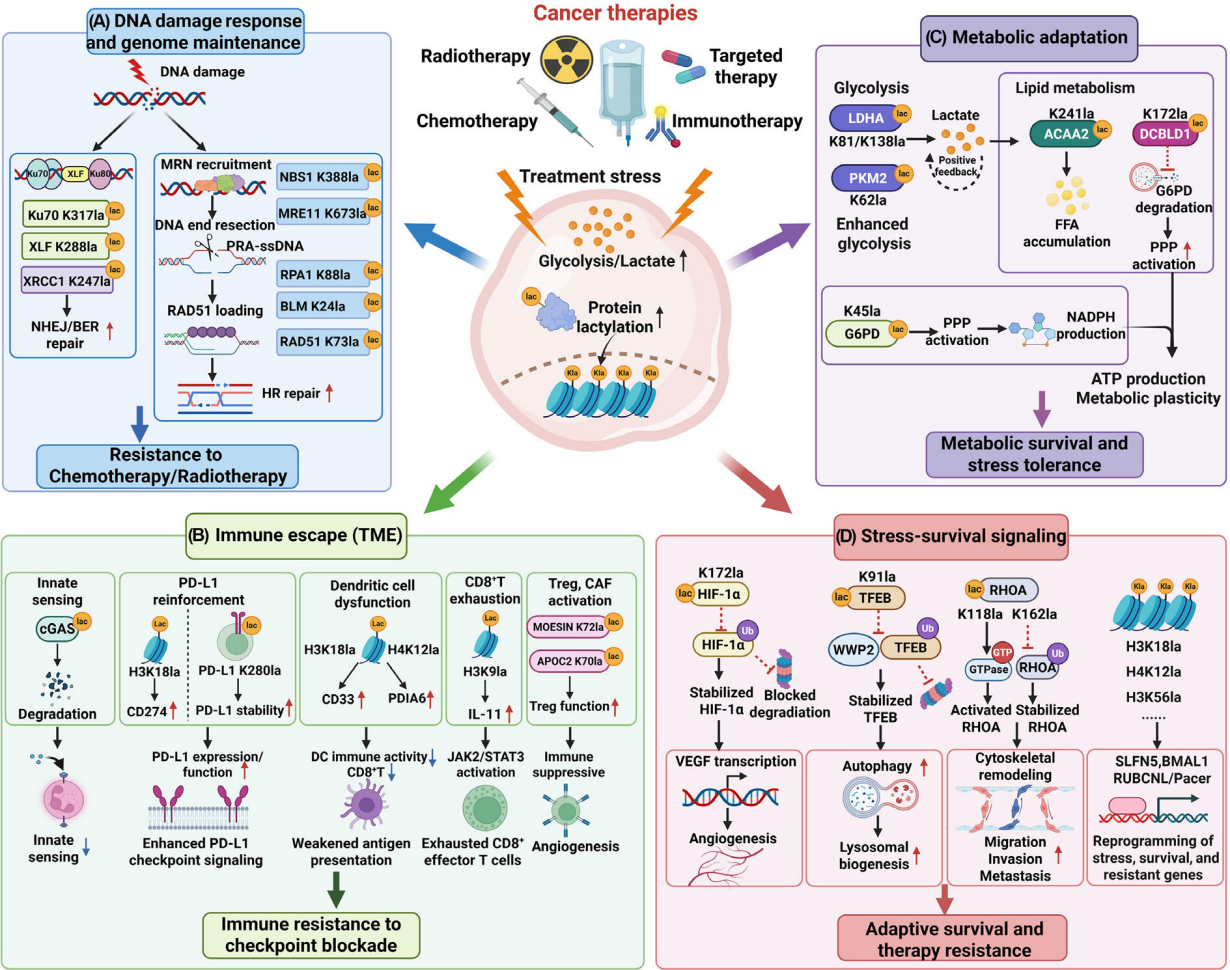
Lactylation‐driven malignant phenotypes and adaptive signaling networks. Cancer therapies—including chemotherapy, radiotherapy, targeted therapy, and immunotherapy—impose convergent treatment stress that elevates glycolytic flux and intracellular lactate, thereby expanding protein lactylation on both histone and nonhistone substrates. (A) DNA damage response and genome maintenance: Treatment‐induced lactate accumulation promotes lactylation of key repair proteins, including components of the NHEJ/BER pathway and the MRN complex, as well as HR mediators such as RAD51. By enhancing repair fidelity at multiple levels, this lactylation program enables tumor cells to tolerate genotoxic insults and underlies resistance to chemotherapy and radiotherapy. (B) Immune escape in the TME: Lactylation suppresses innate immune sensing, reinforces PD‐L1 checkpoint signaling, impairs dendritic cell antigen presentation, and promotes CD8^+^ T cell exhaustion, while simultaneously activating immunosuppressive regulatory T cells and cancer‐associated fibroblasts. This coordinated remodeling of the immune landscape drives resistance to checkpoint immunotherapy. (C) Metabolic adaptation: Lactylation of glycolytic enzymes and lipid metabolic regulators creates positive feedback loops that sustain enhanced glycolysis, redirect carbon flux toward the pentose phosphate pathway, and amplify NADPH and ATP production. These interconnected modifications collectively support metabolic plasticity and stress tolerance under therapeutic pressure. (D) Stress‐survival signaling: Lactylation stabilizes HIF‐1α to drive angiogenesis, activates TFEB‐dependent autophagy and lysosomal biogenesis, promotes RHOA‐mediated cytoskeletal remodeling and metastasis, and reprograms histone marks to induce stress‐adaptive gene expression programs. Together, these outputs converge on adaptive survival and broad therapy resistance. Created with BioRender.com. Abbreviations: FFA, free fatty acid(s); GTPase, guanosine triphosphatase; Ub, ubiquitin; WWP2, WW domain‐containing E3 ubiquitin protein ligase 2.

### 3.1 Chemotherapy‐Induced Protein Lactylation Dynamics

Chemotherapy remains a major component of systemic cancer treatment. Its biological effects extend beyond acute tumor cell killing. In surviving cells, chemotherapeutic stress drives metabolic rewiring characterized by increased glycolytic dependence, elevated lactate production, and expansion of histone or nonhistone lactylation in treatment‐exposed or drug‐resistant populations [31, 116, 117, 118, 119]. The central question is whether chemotherapy generates a metabolism–lactylation axis that helps tumor cells persist. Current evidence supports this idea most clearly in the DDR, with additional support from histone lactylation‐associated resistance circuits and emerging noncanonical mechanisms involving RNA metabolism and tumor–stroma crosstalk.

#### 3.1.1 DNA Repair as the Primary Resistance Interface

Among chemotherapy‐associated contexts, the strongest evidence for a functional role of lactylation lies in the DDR. Genotoxic agents such as cisplatin impose strong selective pressure on tumor cells to maintain repair competence [120]. Recent studies indicate that this pressure can be coupled to lactate‐dependent regulation of repair machinery. In GC models, chemotherapy‐associated metabolic stress increases lactate availability and promotes NBS1 K388 lactylation. This modification facilitates NBS1 recruitment to DNA break sites, enhances assembly of the MRE11–RAD50–NBS1 (MRN) complex, and improves homologous recombination (HR) efficiency, thereby increasing tolerance to platinum‐based treatment [13, 39, 119].

Interventional studies support this pathway's relevance. Genetic or pharmacological suppression of lactate production, including LDHA inhibition with stiripentol, reduces NBS1 lactylation, compromises repair capacity, and resensitizes tumor cells to cisplatin [39]. Related studies suggest that lactylation‐dependent regulation extends to other repair‐associated proteins. MRE11 K673 lactylation enhances DNA binding affinity and end resection efficiency by stabilizing the MRE11–DNA interaction interface, thereby promoting HR repair [13]. This modification proves particularly important in BRCA1‐deficient tumors, where enhanced MRE11 activity partially compensates for impaired HR initiation. Although the full substrate spectrum remains incompletely defined, current evidence supports a clear conclusion: in at least some chemotherapy settings, lactylation functions as an active regulator of repair competence rather than merely reflecting metabolic stress.

#### 3.1.2 Histone Lactylation and Transcriptional Reprogramming

Chemotherapy‐associated lactylation may also reinforce resistance through chromatin‐level transcriptional reprogramming. Histone lactylation connects lactate‐rich stress states with changes in promoter accessibility and gene expression [89, 90, 121, 122]. A well‐defined example comes from HCC. Oxaliplatin‐ and 5‐fluorouracil‐resistant cells show enhanced glycolytic activity together with increased global lactylation, with H3K14la identified as a prominent regulatory mark [117]. Mechanistically, H3K14la promotes transcription of the E3 ubiquitin ligase NEDD4. NEDD4 accelerates PTEN ubiquitination and degradation. PTEN loss further enhances glycolytic signaling, forming a reinforcing loop linked to resistance to both oxaliplatin and 5‐fluorouracil [117].

This model links metabolic rewiring, histone lactylation‐dependent transcription, altered proteostasis, and multidrug resistance within a single framework. However, it has been defined mainly in HCC and should be interpreted as a strong tumor‐specific example rather than a universal mechanism across chemotherapy‐treated cancers [123].

#### 3.1.3 RNA Metabolism and Microenvironmental Lactylation

Beyond DNA repair and histone‐mediated transcriptional control, emerging evidence suggests that chemotherapy‐associated lactylation may also influence resistance through RNA processing and tumor–stroma metabolic interactions. One example involves METTL3, an m^6^A methyltransferase whose lactylation has been implicated in cisplatin response in TNBC. METTL3 lactylation regulates m^6^A‐associated processing of DDR transcripts, suggesting that tumor cells may use lactylation not only to support repair or reshape chromatin output, but also to modulate RNA metabolism during chemotherapy stress [124].

A recent study in colorectal cancer extends this framework to the TME. In oxaliplatin‐resistant colorectal cancer, glycolytic cancer‐associated fibroblasts (CAFs) produce lactate that promotes ANTXR1 transcription through histone lactylation in cancer cells and induces ANTXR1 K453 lactylation, increasing ANTXR1 protein stability [125]. This dual program increases ANTXR1 expression and protein stability, activates the RhoC/ROCK1/SMAD5 pathway, strengthens stem‐like properties, and promotes oxaliplatin resistance. Importantly, genetic or pharmacological blockade of the lactate shuttle between fibroblasts and cancer cells improves oxaliplatin efficacy in vitro and in cell‐ and patient‐derived xenograft models [125]. This study demonstrates that chemotherapy‐associated lactylation can be driven not only by tumor cell‐intrinsic metabolism, but also by stromal lactate transfer.

#### 3.1.4 Potential Biomarkers and Therapeutic Targets

The evidence reviewed above supports protein lactylation as a functional contributor to chemotherapy resistance, though the strength and breadth of evidence vary across mechanisms and tumor types. Evidence remains uneven across drug classes and tumor contexts. In anthracycline‐, antimetabolite‐, and taxane‐resistant settings, resistant cells often display glycolytic reinforcement or lactate‐rich phenotypes, but the causal contribution of specific lactylation events remains incompletely resolved [117, 118, 126, 127, 128]. Proposed phenomena such as persistent pan‐lactylation after drug withdrawal, lactylation‐associated metabolic memory, or mitotic stress‐linked lactylation changes remain mechanistically interesting but are not yet established as general principles [115, 124, 129]. This raises two translational questions: Can protein lactylation serves as a predictive or prognostic biomarker? Can it be targeted to restore drug sensitivity?

Biomarker potential appears strongest for histone marks such as H3K18la and H3K14la. These marks correlate with poor response to chemotherapy in hepatocellular, gastric, and colorectal cancers [31, 117, 130, 131]. Site‐specific lactylation of repair proteins (e.g., NBS1 K388la, MRE11 K673la) may also predict platinum resistance in selected contexts [13, 39]. However, clinical validation remains limited. Most studies rely on preclinical models or small retrospective cohorts. Prospective trials incorporating lactylation profiling at baseline and during treatment are needed to establish clinical utility.

Therapeutic targeting is more advanced conceptually than clinically. Strategies include: (i) reducing lactate production through LDHA inhibition (e.g., stiripentol, oxamate) [39, 117]; (ii) blocking lactate transport via MCT1/4 inhibitors (e.g., AZD3965) [132]; (iii) inhibiting lactylation writers such as p300/CBP (e.g., A‐485, CCS1477) [6, 133, 134]; (iv) Enhancing delactylation through HDAC activation or sirtuin modulation [10, 72, 79, 135].

Clinical translation faces several challenges. Protein lactylation is not uniformly elevated across all chemotherapy‐resistant tumors [117, 123]. Its contribution may vary by tumor type, metabolic state, and treatment regimen [136]. Targeting protein lactylation may also affect normal tissue repair and immune function [6, 60, 137]. These considerations suggest that lactylation‐targeted interventions should be pursued in biomarker‐selected populations rather than as broad‐spectrum strategies.

### 3.2 Radiotherapy‐Induced Protein Lactylation Dynamics

Radiotherapy induces acute DNA damage and oxidative injury, which are central to tumor control [138, 139, 140]. In surviving cells, irradiation can also cause mitochondrial dysfunction, reactive oxygen species (ROS) accumulation, transient hypoxia, and compensatory glycolysis, thereby increasing lactate availability and favoring histone and nonhistone protein lactylation [141, 142, 143, 144]. These metabolic and redox changes may further shift the lactylation–delactylation balance through Class I HDACs and NAD^+^‐dependent sirtuins. Consequently, protein lactylation may support DNA repair, redox recovery, and stress resilience after irradiation. However, whether HDACs or sirtuins directly regulate tumor radiosensitivity through their delactylase activities remains unresolved.

#### 3.2.1 Histone Lactylation and Chromatin‐Level Repair Priming

One early consequence of radiation‐associated metabolic stress is the formation of a chromatin environment that permits more efficient DNA damage signaling and repair. As lactate accumulates, it supports histone lactylation through increased lactyl‐donor availability and through changes in the acylation‐deacylation balance controlled by enzymes such as p300/CBP and selected deacylases [141, 142, 145, 146]. In irradiated tumor models, increased H3K18la has been linked to enhanced chromatin accessibility and more efficient access of repair factors to damaged DNA [89, 147].

This effect can be understood as chromatin‐level repair priming. Radiation‐associated H3K18la connects metabolic disturbance with a nuclear state that is more permissive for repair machinery assembly. Beyond facilitating repair factor access, H3K18la may also drive transcriptional programs that sustain radioresistance. In glioblastoma stem cells, H3K18 lactylation upregulates USP4, which stabilizes ANXA2 and activates STAT3 signaling to support stem cell maintenance and radioresistance [148]. In HCC, histone lactylation promotes MCM7 transcription, enhancing stemness and reducing radiation sensitivity [29]. This is not a passive reflection of lactate accumulation but rather an active mechanism by which irradiated tumor cells convert short‐term metabolic stress into a repair‐supportive chromatin configuration and preserve stem‐like features [147, 149, 150]. This chromatin‐level effect cooperates with direct lactylation of DNA repair proteins themselves, which is detailed in the following section.

#### 3.2.2 Enhancement of DNA Repair Pathways

Beyond chromatin‐level priming, the most substantial mechanistic evidence in the radiotherapy setting concerns direct lactylation of DNA repair proteins. Lactylation of HR factors contributes to radiotherapy resistance. In glioblastoma, HAT1 functions as a lactyltransferase that modifies RPA1 at K88, promoting DNA repair. Lactate enhances both HAT1 expression and enzymatic activity. HAT1 depletion or inhibition of RPA1 lactylation improves radiotherapy efficacy in glioblastoma [151].

More specifically for radiotherapy, lactylation supports repair through pathways beyond HR. In nonhomologous end joining (NHEJ), XLF lactylation strengthens its interaction with Ku80 and promotes more efficient recruitment to DNA double‐strand breaks (DSBs) [152, 153, 154]. In parallel, XRCC1 lactylation promotes nuclear localization and accumulation of repair factors involved in base excision repair (BER) and single‐strand break repair (SSBR). This process is linked to tolerance to chemoradiotherapy in highly glycolytic tumor states [150, 155]. These findings support a broader conclusion: under irradiation, the lactate–lactylation axis strengthens repair competence across multiple branches of the DDR, reducing radiosensitivity in metabolically flexible tumor cells.

#### 3.2.3 Redox Homeostasis and Cell Fate Control

Radiotherapy‐induced protein lactylation may also support survival through mechanisms beyond direct DNA repair. Ionizing radiation generates strong oxidative stress. Residual tumor cells must restore redox balance quickly to avoid lethal injury. Current evidence indicates that elevated lactylation affects metabolic enzymes involved in NADPH‐generating flux, antioxidant defense, and broader stress‐buffering programs. These changes help reduce the impact of radiation‐induced ROS and promote metabolic recovery after irradiation [112, 156, 157, 158, 159].

A related layer involves cell‐fate control. In selected tumor models, H3K18la and H4K12la have been linked to repression of proapoptotic programs and reinforcement of antiapoptotic signaling networks, including Bcl‐2 family pathways [98, 160, 161, 162, 163]. Additional studies show that lactylation may intersect with autophagy‐ and ferroptosis‐related responses under radiotherapy‐associated stress. For example, Vps34 K356/K781 lactylation catalyzed by KAT5/TIP60 enhances autophagosome formation and lysosomal degradation. This modification has been reported to enhance autophagic flux by promoting autophagosome formation and lysosomal degradation [164, 165].

These mechanisms expand the role of protein lactylation beyond repair. They suggest that radiation‐induced protein lactylation can bias stressed tumor cells away from irreversible death and toward recovery, persistence, or senescence escape. The exact outcome depends on tumor lineage, oxygenation state, radiation dose, and treatment schedule.

#### 3.2.4 Radiosensitization Strategies Through Targeting Protein Lactylation

Radiotherapy studies support protein lactylation as a functional mediator of radioadaptive survival in defined experimental contexts. The strongest rationale comes from evidence that repair proteins can be enhanced by lactylation in lactate‐rich or glycolytically active tumor states. In parallel, histone lactylation contributes to chromatin accessibility and repair‐permissive nuclear organization [13, 119, 149, 150, 152]. These findings suggest that tumors with strong glycolytic adaptation during or after irradiation may enter a lactylation‐supported repair state that reduces treatment sensitivity.

This model provides a practical therapeutic direction. Interventions that lower lactate production, block lactate transport, or suppress specific lactylation events may sensitize tumor cells to radiotherapy by impairing both DNA repair and stress resilience [157, 166]. As detailed in Section 3.1.4, general strategies include LDHA inhibition (e.g., stiripentol) and MCT1/4 blockade (e.g., AZD3965) [167, 168]. In the radiotherapy context, additional radiotherapy‐specific strategies have emerged: (i) blocking the ALDH1A3–PKM2 interaction (e.g., D34‐919) to suppress XRCC1 lactylation and restore sensitivity to chemoradiotherapy in glioblastoma [150, 169]; (ii) lactate scavenging using biomaterials (e.g., CoMnFe–LDO nanosheets that catalyze lactate conversion to pyruvate), which depletes lactate in the TME and enhances radiotherapy‐induced ROS generation [170]. A mild‐hyperthermia‐guided radiosensitizer, GQD@PDA/Au, has also been reported to reduce lactate levels, exacerbate DNA damage, and enhance tumor radiosensitivity [171].

Clinical translation remains early. Most evidence comes from preclinical systems [146]. The contribution of histone versus nonhistone lactylation may differ across tumor types, radiation schedules, and oxygenation states [89, 90, 147]. The central message is not that all irradiated tumors become lactylation‐driven. Rather, radiotherapy can generate, in selected contexts, a metabolic–lactylation radioadaptive axis that has now been supported by several site‐resolved and pathway‐level studies.

### 3.3 Targeted Therapy‐Induced Protein Lactylation Dynamics

Targeted therapy differs from chemotherapy and radiotherapy. It imposes a more selective, pathway‐defined stress. Many targeted agents—particularly tyrosine kinase inhibitors (TKIs)—initially suppress the oncogenic signaling networks that sustain glycolysis, biosynthesis, and survival [172, 173, 174, 175]. Unlike the immediate metabolic activation seen with cytotoxic therapies (Sections 3.1 and 3.2), the relationship between targeted therapy and protein lactylation follows a biphasic pattern. In several treatment contexts, early response to pathway blockade is accompanied by reduced glycolytic output and lower lactate availability. Prolonged exposure, however, can promote compensatory metabolic rewiring and re‐emergence of lactylation‐dependent states [43, 176, 177, 178, 179]. This temporal pattern is central to acquired resistance. Protein lactylation may be weakened during the early response phase but re‐emerge as a secondary layer during resistant outgrowth.

#### 3.3.1 Early Metabolic Suppression

The initial effect of successful targeted therapy is often metabolic contraction rather than metabolic escalation. In tumors that depend strongly on oncogenic drivers such as BCR–ABL or EGFR, blockade of these pathways can rapidly reduce glycolytic flux. This occurs by suppressing downstream PI3K/AKT/mTOR, MYC‐associated, or related anabolic signaling programs [173, 177, 180, 181, 182]. In chronic myeloid leukemia, imatinib has been reported to reduce expression of glycolysis‐associated enzymes such as HK2 and LDHA. Similarly, in EGFR‐mutant NSCLC, osimertinib can suppress core glycolytic activity. It may also reduce the membrane abundance or functional contribution of lactate transporters such as MCT1/4. This further limits lactate exchange in treatment‐sensitive cells [43, 45, 176, 183, 184].

These early events are important because they demonstrate that protein lactylation is not inevitably elevated under all therapeutic conditions. In targeted therapy, at least during the initial response window, effective signal blockade may temporarily interrupt the metabolic–lactylation axis rather than activate it. This feature distinguishes targeted therapy from the more immediate damage‐associated stress responses seen with cytotoxic treatment. It helps explain why protein lactylation in TKI‐treated tumors should be viewed as a dynamic variable rather than a fixed resistance marker.

#### 3.3.2 Protein Lactylation Re‐Emergence and Bypass Signaling

The situation changes as resistance develops. Residual tumor cells that survive continued pathway inhibition frequently restore glycolytic capacity through compensatory rewiring, alternative nutrient use, or bypass activation of parallel signaling pathways [43, 176, 185, 186]. Once lactate homeostasis is re‐established, protein lactylation may re‐enter the resistance program as a mechanism that stabilizes evasive signaling and supports drug‐tolerant phenotypes. Evidence from osimertinib‐resistant lung adenocarcinoma suggests that lactate metabolism can feed back into the resistance network through ENO1‐associated lactylation circuitry. This links metabolic restoration to persistent signaling [184]. Additional work has shown that NNMT can promote acquired EGFR‐TKI resistance by forming an EGR1–lactate double‐positive feedback loop. This further underscore the role of lactate‐centered metabolic rewiring in sustaining resistance states [187].

A more concrete example of lactylation‐dependent protein stabilization is provided by DCBLD1. In cervical cancer, lactate increases HIF‐1α enrichment at the DCBLD1 promoter. It also induces DCBLD1 K172 lactylation. This stabilizes the protein and prolongs its half‐life [112]. Functionally, stabilized DCBLD1 inhibits autophagic degradation of G6PD, activates the PPP, and promotes tumor proliferation and metastasis [112]. Although this mechanism was not defined in a classic TKI‐resistance model, it establishes an important principle: lactylation can stabilize signaling‐ or scaffold‐associated proteins and redirect metabolic flux toward survival‐supporting pathways, helping resistant cells maintain redox balance and biosynthetic capacity despite continued pathway inhibition.

#### 3.3.3 Metabolic Reprogramming and Drug Tolerance

Targeted‐therapy resistance is not maintained by signaling bypass alone. Lactylation may also participate in epigenetic and posttranscriptional programs that allow tumor cells to survive prolonged pathway inhibition. In resistant tumor states, elevated lactate has been associated with increased histone lactylation at loci linked to drug tolerance, stemness, and survival maintenance [29, 101, 116, 117, 162]. Although the specific loci and functional outputs vary across tumor types, these findings support the idea that protein lactylation can help lock transient stress responses into more durable drug‐tolerant states.

A well‐characterized example involves lenvatinib resistance in HCC. In this setting, enhanced glycolysis and lactate accumulation drive IGF2BP3 K76 lactylation, which promotes m^6^A‐related regulation of PCK2 and NRF2 transcripts, reprograms serine metabolism, and strengthens antioxidant defense [188]. Mechanistically, lactylated IGF2BP3 captures PCK2 and NRF2 mRNAs for m^6^A modification, while altered serine metabolism increases methylated substrates (e.g., S‐adenosylmethionine) to reinforce the antioxidant system. HECTD2 lactylation has also been implicated in lenvatinib resistance by promoting KEAP1 degradation and activating the KEAP1/NRF2 pathway [189]. Notably, H3K18la promotes HECTD2 transcription, creating a lactate–lactylation–antioxidant reinforcing loop. Together, these findings indicate that protein lactylation may help maintain drug‐tolerant states by coordinating transcriptional, posttranscriptional, and metabolic survival programs.

#### 3.3.4 The Hypoxia–Lactate–Lactylation Axis in Antiangiogenic Resistance

The role of lactylation in targeted therapy is especially prominent in antiangiogenic treatment. Unlike oncogene‐directed TKIs, antiangiogenic agents intensify hypoxia, alter nutrient gradients, and create a microenvironment that favors glycolytic compensation and lactate accumulation [56, 131, 190, 191, 192]. This setting is highly conducive to protein lactylation remodeling, as antivascular pressure selects for tumor cells that can survive under hypoxic, acidic, and metabolically constrained conditions.

The clearest example comes from colorectal cancer. Tumor‐derived lactate promotes bevacizumab resistance by increasing P300‐catalyzed H3K18la at the RUBCNL locus. This upregulates RUBCNL/Pacer, enhances autophagosome maturation, and supports survival under anti‐VEGF pressure [131]. This mechanism directly links therapy‐induced microenvironmental stress, histone lactylation, autophagy, and evasive resistance. In this context, protein lactylation is not only a marker of a lactate‐rich tumor state. It also helps create an autophagy‐permissive transcriptional program that buffers vascular and nutrient restriction.

Other studies suggest that antiangiogenic resistance can be supported by several convergent lactylation‐dependent routes. In ovarian cancer, ENO1 lactylation has been reported to drive bevacizumab resistance through metabolic reprogramming and angiogenesis [193]. In glioblastoma, lactate‐induced protein lactylation promotes BMAL1 transcription and nuclear translocation, enhancing VEGFA signaling and bevacizumab resistance [194]. In colorectal cancer, PKM2 lactylation promotes vasculogenic mimicry and bevacizumab resistance by facilitating FOSL1 super‐enhancer formation [195]. These findings suggest that antiangiogenic therapy can activate a hypoxia–lactate–lactylation escape axis involving autophagy enhancement, proangiogenic transcriptional recovery, metabolic reprogramming, and vascular mimicry‐like compensation.

Among targeted‐therapy contexts, antiangiogenic treatment may therefore represent one of the strongest examples of lactylation‐driven resistance. The initiating pressure is vascular and metabolic restriction. The intermediate response is hypoxia‐driven glycolytic rewiring and lactate accumulation. The downstream mechanism is histone and nonhistone lactylation remodeling. The final output is improved survival through autophagy, angiogenic compensation, or vasculature‐independent escape programs.

#### 3.3.5 Temporal Dynamics and Intervention Windows

Current evidence supports a temporal and state‐dependent model of protein lactylation in targeted therapy. During the initial drug‐sensitive phase, effective oncogenic blockade may reduce glycolysis and weaken the protein lactylation landscape [173, 187, 196]. During the resistant phase, tumor cells often restore lactate metabolism, allowing protein lactylation to reappear as a mediator of bypass signaling and persistent survival [43, 45, 178, 197]. In this sense, protein lactylation functions less as an immediate consequence of target inhibition and more as a secondary layer that helps convert partial pathway escape into stable resistance.

This temporal pattern suggests distinct intervention windows. During the early phase (0–4 weeks), monitoring lactylation suppression may serve as a response indicator; premature metabolic intervention could be counterproductive [173, 196, 198, 199, 200]. During the transition phase (4–12 weeks), detecting protein lactylation re‐emergence may signal early resistance; this is the optimal window to initiate combination therapy [178, 201, 202, 203, 204]. During the resistant phase (>12 weeks), targeting established lactylation‐dependent pathways (e.g., H3K18la–HECTD2–NRF2 axis, H3K18la–RUBCNL autophagy circuit, ERK K231la–RAS pathway) become necessary [131, 189, 205].

However, the evidence base remains heterogeneous across tumor types and drug classes. Mechanisms such as H3K18la–HECTD2 signaling in lenvatinib resistance and H3K18la–RUBCNL signaling in bevacizumab resistance are supported by relatively direct experimental evidence, whereas other mechanisms remain more preliminary [131, 189]. The most defensible conclusion is that targeted therapy can generate a dynamic metabolic–lactylation cycle, in which early suppression is followed by resistance‐associated reactivation. This cycle may provide a tractable vulnerability in selected tumor contexts.

### 3.4 Immunotherapy‐Induced Protein Lactylation Dynamics

Immunotherapy differs from chemotherapy, radiotherapy, and targeted therapy in that its efficacy depends less on direct tumor cell killing and more on the immune state of the TME. In this setting, lactate accumulation and protein lactylation have emerged as important links between tumor metabolic reprogramming and immune escape [46, 47, 52, 59]. Current evidence suggests that lactylation acts across both tumor cells and multiple immune cell populations, thereby reshaping checkpoint signaling, antigen presentation, effector T‐cell activity, and myeloid polarization [52, 59, 206]. Through these combined effects, protein lactylation may help establish a TME that is less responsive to immune checkpoint blockade.

#### 3.4.1 PD‐L1 Reinforcement in Tumor Cells

In tumor cells, one of the most prominent immunotherapy‐related effects of protein lactylation is the enhancement of PD‐L1 expression or function. Histone lactylation, notably H3K18la (as elaborated in Section 3.1.2), has been associated with the transcriptional upregulation of CD274 (the gene encoding PD‐L1) across various tumor types, including hepatocellular, gastric, and colorectal cancers, frequently mediated by regulators such as c‐MYC [59, 93, 94, 207, 208]. Concurrently, nonhistone lactylation can exert a more direct influence on PD‐L1 at the protein level. Specifically, lactylation at PD‐L1 K280 enhances protein stability by mitigating ubiquitin‐dependent degradation [51], whereas delactylation at K189 facilitates its nuclear translocation and augments tumor‐promoting transcriptional activity [209]. Collectively, these observations suggest that lactylation can potentiate immune evasion mechanisms related to checkpoint inhibition through both transcriptional and posttranslational pathways. This intrinsic tumor checkpoint reinforcement is likely to synergize with lactylation‐induced dysfunction in adjacent immune cells.

#### 3.4.2 Dendritic Cell Dysfunction

Dendritic cells are essential for antigen presentation and T‐cell priming, and their dysfunction can substantially weaken the response to immune checkpoint inhibitors [210, 211, 212, 213]. Recent studies suggest that protein lactylation can maintain dendritic cells in a poorly stimulatory state. In colorectal cancer, excess lactate driven by MPC dysregulation increases H3K18 lactylation and is associated with an immature dendritic‐cell program marked by CD33 upregulation, reduced support for CD8^+^ T‐cell function, and weaker anti‐PD‐1 efficacy [214]. In GC, endoplasmic reticulum stress induces H4K12 lactylation, promotes PDIA6 expression in dendritic cells, and attenuates their immune activity [215]. These findings support a context‐dependent role for protein lactylation in weakening dendritic‐cell immunostimulatory capacity.

#### 3.4.3 T‐Cell Exhaustion and Treg Expansion

Lactylation‐associated immune remodeling also affects lymphoid compartments. In CD8^+^ T cells, elevated protein lactylation has been associated with impaired cytotoxicity and dysfunctional or exhaustion‐like states [49, 216]. One representative mechanism is the H3K9la–IL‐11–JAK2/STAT3 axis described in head and neck squamous cell carcinoma, which weakens CD8^+^ T‐cell antitumor activity and is linked to poor immunotherapy response. At the same time, protein lactylation appears to support suppressive lymphoid populations. Nonhistone lactylation events involving MOESIN K72 and APOC2 K70 have been reported to enhance Treg stability, accumulation, or function, thereby reinforcing immune suppression and resistance to anti‐PD‐1 treatment [217, 218]. Notably, APOC2 K70 lactylation upregulates immunosuppressive pathways in Tregs, promoting their suppressive function and limiting antitumor immunity. Overall, protein lactylation seems to shift lymphoid immunity away from effective tumor control and toward suppressive dominance.

#### 3.4.4 Myeloid Immunosuppressive Reprogramming

Among immune cell populations, myeloid cells currently provide some of the strongest evidence for lactylation‐dependent immunosuppression. Histone lactylation has been repeatedly associated with macrophage polarization toward tumor‐supportive and anti‐inflammatory states, including induction of genes linked to reparative or M2‐like programs [26, 219, 220]. Nonhistone lactylation further stabilizes this phenotype. A representative example is METTL3 lactylation, which enhances Jak1 translation and amplifies JAK1/STAT3 signaling in tumor‐infiltrating myeloid cells, thereby suppressing proinflammatory activity [58]. Additional work suggests that protein lactylation may also weaken innate immune sensing pathways such as RIG‐I in selected tumor contexts [18, 34, 221].

#### 3.4.5 Therapeutic Strategies Targeting Lactylation in Immunotherapy

Current evidence supports protein lactylation as an active regulator of immunotherapy response. By reinforcing PD‐L1 signaling, weakening dendritic‐cell function, impairing CD8+ T‐cell activity, supporting Treg persistence, and stabilizing suppressive myeloid states, lactylation contributes to a TME that is less permissive to checkpoint blockade [58, 59, 214, 217, 222]. Although the exact substrates and downstream pathways vary across tumor types, the overall pattern is consistent: protein lactylation favors immune escape and treatment resistance.

Metabolic interventions that reduce lactate accumulation (e.g., LDHA inhibition, MCT1/4 blockade) may act as broad‐spectrum immune reactivators by simultaneously targeting multiple lactylation‐dependent immunosuppressive pathways [221, 223, 224]. However, clinical translation requires careful consideration of timing and patient selection. Prospective trials incorporating protein lactylation profiling at baseline and during treatment will be essential to identify patients most likely to benefit from lactylation‐targeted immunotherapy combinations.

In summary, current evidence demonstrates that protein lactylation functions as a dynamic integrator of treatment‐induced stress across chemotherapy, radiotherapy, targeted therapy, and immunotherapy. Although each modality imposes distinct metabolic pressures, protein lactylation consistently emerges as a common resistance mechanism linking DNA repair, metabolic reprogramming, and immune evasion. These treatment‐specific protein lactylation dynamics raise a fundamental question: what are the underlying biological processes through which protein lactylation establishes and maintains therapy‐resistant states? The following section systematically examines how protein lactylation coordinates key cancer hallmarks—from DDR and metabolic rewiring to microenvironmental remodeling—to reveal the mechanistic principles that position this metabolic–epigenetic switch as a targetable vulnerability in cancer therapy.

## 4 Molecular Mechanisms of Protein Lactylation in Regulating Cancer Therapy

Therapy resistance remains a central challenge in cancer treatment. Tumor cells evade therapeutic pressure through multiple interconnected mechanisms, including enhanced DNA repair, redox rebalancing, cell‐cycle checkpoint evasion, sustained proliferative signaling, metabolic flexibility, microenvironment remodeling, and cancer stem cell persistence. Protein lactylation connects these processes by converting lactate accumulation into functional changes in chromatin structure, transcriptional output, and protein activity. This section examines how protein lactylation contributes to treatment resistance across seven key biological domains, with emphasis on substrate‐specific mechanisms and their therapeutic implications.

### 4.1 Protein Lactylation in DNA Damage Repair Regulation

DNA damage repair is a dominant mechanism by which protein lactylation influences therapy response. Platinum agents, anthracyclines, PARP inhibitors, and radiotherapy induce lethal DNA lesions; their efficacy depends on overwhelming the tumor cell's repair capacity. Lactylation shifts this balance by selectively tuning specific repair nodes to match lesion type and therapeutic pressure [13, 16, 39, 118] (Figure 4A).

**FIGURE 4:**
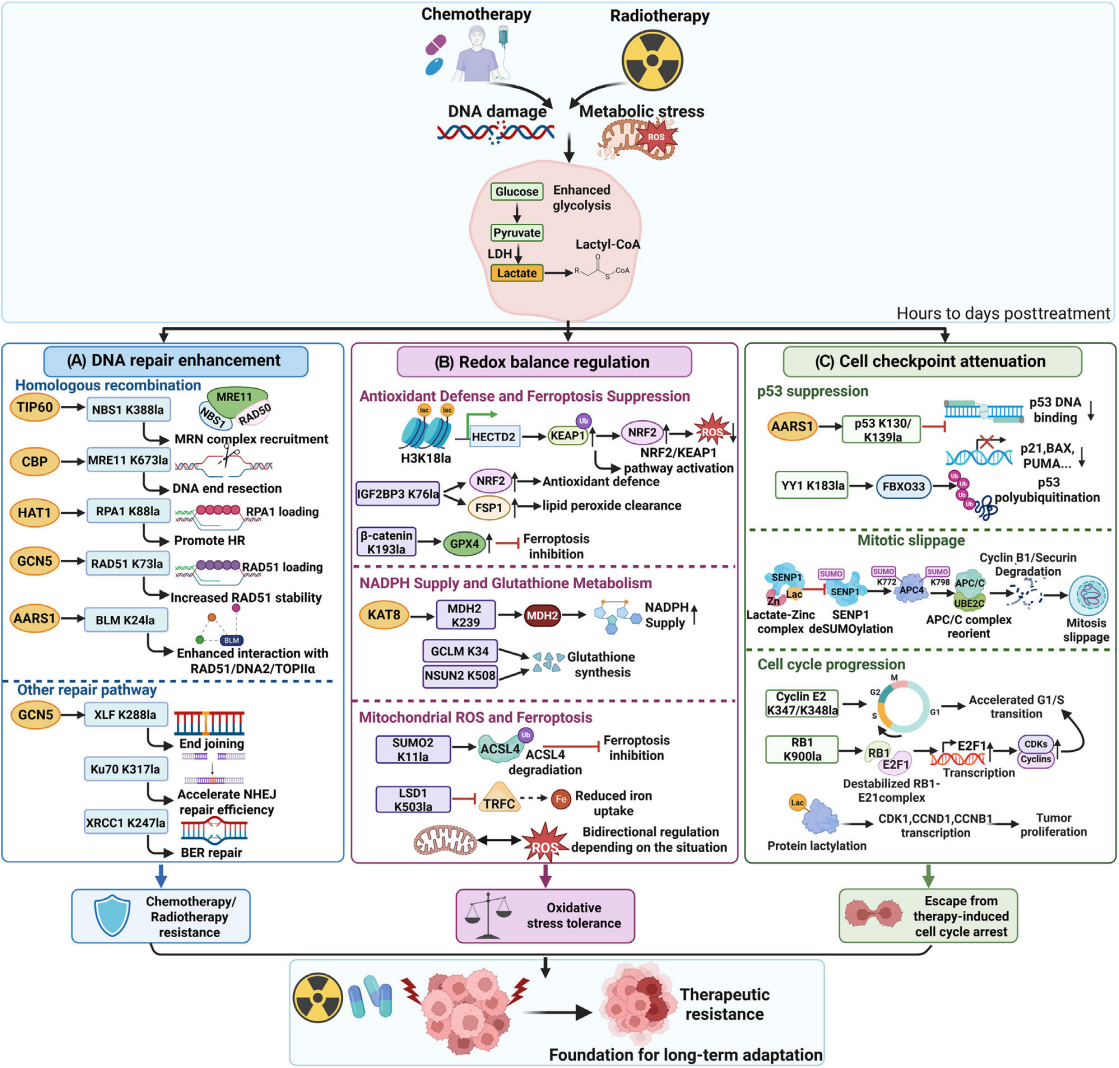
Lactylation‐mediated tumor survival under early therapeutic stress. Therapeutic interventions including chemotherapy, radiotherapy, and targeted therapy induce acute metabolic stress and DNA damage in tumor cells, driving enhanced glycolysis and lactate accumulation. Elevated lactate is converted to lactyl‐CoA and catalyzes protein lactylation through lactyltransferases (TIP60, CBP, GCN5, AARS1). Three parallel survival pathways are activated: (A) DNA repair enhancement—lactylation of homologous recombination factors (NBS1 K388la, MRE11 K673la, RAD51 K73la, BLM K24la) and base excision repair proteins (XRCC1 K247/K256la) strengthens DNA damage response capacity, conferring chemotherapy and radiotherapy resistance; (B) redox balance regulation—H3K18la activates the NRF2/KEAP1 antioxidant pathway, while SUMO2 K11la promotes ACSL4 degradation and LSD1 K503la reduces TFRC expression, coordinately suppressing ferroptosis and enabling oxidative stress tolerance; (C) cell cycle checkpoint attenuation—AARS1‐mediated p53 K120/K139la impairs p53 transcriptional activity, H3K18la upregulates MDM2 to accelerate p53 degradation, PLK1 activity maintenance promotes mitotic slippage, and lactylation of cyclins (CCNE2 K347/K348la) and histone‐mediated upregulation of cell cycle genes (CCNB1/CDK1/CCND1) drive cell cycle progression despite therapeutic pressure. These convergent mechanisms enable tumor cell survival during the acute phase of treatment (hours to days), establishing a foundation for subsequent long‐term adaptation. Abbreviations: BAX, BCL2‐associated X protein; CDKs, cyclin‐dependent kinases; DNA2, DNA replication helicase/nuclease 2; p21, cyclin‐dependent kinase inhibitor 1A; PUMA, p53 upregulated modulator of apoptosis; SUMO, small ubiquitin‐like modifier; TopIIα, DNA topoisomerase II alpha.

#### 4.1.1 HR Enhancement

HR requires coordinated damage sensing, MRN complex assembly, DNA end resection, RAD51 loading, and strand exchange. Protein lactylation reinforces this pathway through both transcriptional activation and functional enhancement of HR proteins [13, 16, 118, 225].

At the chromatin level, histone lactylation drives HR gene expression. Elevated H3K9la directly transactivates core HR genes such as RAD51 and BRCA2, enhancing DSB repair efficiency [119]. While H4K12la enrichment at the RAD23A super‐enhancer region facilitates MYC recruitment, amplifying HR capacity and promoting resistance. This epigenetic layer increases the availability of repair machinery under genotoxic stress [226]. At the protein level, NBS1 K388la (catalyzed by TIP60, reversed by HDAC3) enhances MRN recruitment to DSBs. in GC patients, high NBS1 K388la correlates with poor response to neoadjuvant chemotherapy [10, 39]. MRE11 K673la (mediated by CBP) increases DNA binding and resection efficiency, conferring tolerance to cisplatin and PARP inhibition [13]. Later HR steps are also lactylation‐sensitive: AARS1‐mediated BLM K24la stabilizes Bloom syndrome protein (BLM) and enhances its interaction with RAD51 in anthracycline‐resistant cells [17, 118]. GCN5‐mediated RAD51 K73la increases RAD51 stability in platinum‐resistant ovarian cancer [118, 119]. HAT1‐catalyzed RPA1 K88la promotes HR‐mediated radioresistance in glioblastoma and lung adenocarcinoma [16, 151].

This two‐tiered network—histone lactylation increasing repair gene transcription and nonhistone lactylation enhancing repair protein function—defines a robust lactylation‐dependent HR system. Tumors with this system may resist DNA‐damaging therapies; conversely, disrupting specific lactylation events (e.g., with HDAC3 or AARS1 inhibitors) can restore sensitivity.

#### 4.1.2 NHEJ and BER/SSBR: Repair Pathway Redundancy

Protein Lactylation also engages alternative repair pathways. In NHEJ, ionizing radiation or chemotherapy activates the ATM–GCN5 axis, promoting XLF K288la lactylation and accelerating end joining [152]. Ku70 K317la strengthens Ku70–Ku80–DNA–PKcs complex formation, boosting NHEJ efficiency and supporting chemoradiotherapy tolerance in glioma stem cells [221]. For BER/SSBR, XRCC1 K247la is induced in ALDH1A3‐high glioblastoma under lactate‐rich conditions [150]. This modification promotes XRCC1 nuclear translocation and enrichment at damage sites, facilitating repair‐complex assembly and lesion clearance. Such activity helps metabolically adapted cells survive radiotherapy or temozolomide [150]. These findings reveal a broader principle: protein lactylation provides repair redundancy. HR‐related protein lactylation ensures high‐fidelity DSB repair; NHEJ‐ and BER/SSBR‐related lactylation handles alternative lesions or intermediates. When one pathway is inhibited (e.g., by PARPi), tumor cells may compensate via lactylation‐enhanced alternatives [227, 228].

#### 4.1.3 Transcriptional and Posttranscriptional Regulation

Beyond direct repair‐protein modification, lactylation also regulates repair gene output. This layer is particularly relevant to acquired resistance, where tumor cells gradually rebuild repair programmes under sustained treatment pressure [6, 229, 230]. At the chromatin level, histone lactylation can either activate or repress specific DNA repair pathways. In glioblastoma, H3K9la‐mediated induction of LUC7L2 suppresses mismatch repair by inhibiting MLH1, contributing to temozolomide resistance. This illustrates how protein lactylation can selectively disable certain repair pathways, creating a compensatory shift toward alternative mechanisms [231]. At the RNA‐regulatory level, lactylation–delactylation intersects with m^6^A modification. In TNBC, HDAC2‐mediated METTL3–K27 delactylation enhances its interaction with Wilms’ tumor 1‐associating protein, increasing METTL3‐dependent m^6^A activity [124]. This improves the stability and translation of repair‐related transcripts, including ASF1B and EXO1, and contributes to acquired cisplatin resistance.

Thus, protein lactylation regulates DNA repair through both protein‐level and gene‐expression‐level mechanisms: direct nonhistone lactylation alters repair‐protein function, while histone lactylation and lactylation‐linked RNA modification modulate repair machinery availability or selectively suppress specific repair pathways.

#### 4.1.4 Writer‐Eraser Balance and Repair Plasticity

DNA repair‐associated protein lactylation is controlled by multiple writers (TIP60, CBP, GCN5, AARS1) and erasers (HDAC3, HDAC2), supporting a substrate‐selective and dynamically regulated protein lactylation network rather than a passive by‐product of lactate accumulation [13, 118, 119, 124, 152]. This regulation enables repair pathway plasticity. Under genotoxic stress, tumor cells can shift pathway usage: favoring HR when resection machinery is lactylated, or switching to NHEJ if Ku70 is modified. Metabolic flux thus couples to repair choice, allowing compensatory responses to therapy‐induced DNA damage. This establishes a “damage‐metabolism‐repair” feedback loop: DNA damage triggers lactate accumulation; lactate drives lactylation of repair proteins; lactylation enhances repair capacity, which protects cells from further damage and sustains metabolic reprogramming [13, 16, 39, 221].

This dynamic interplay suggests potential therapeutic windows. Early‐phase protein lactylation may be transient and reversible, whereas sustained protein lactylation under chronic treatment pressure can lock in stable resistance programs. Targeting protein lactylation during the early adaptation phase may prevent the establishment of resistance rather than attempting to reverse it after it is entrenched.

### 4.2 Protein Lactylation and Oxidative Stress

Oxidative stress is a common consequence of cancer therapy. Radiotherapy, cytotoxic agents, targeted drugs, and ferroptosis‐inducing strategies all increase ROS, lipid peroxidation, or iron‐dependent oxidative injury [140, 232, 233, 234, 235]. Protein lactylation modifies treatment response by regulating antioxidant transcription, iron handling, NADPH supply, glutathione metabolism, and ferroptosis sensitivity [158, 188, 189, 236, 237]. Its effect depends on the modified substrate, the pathway involved, and the oxidative state of the tumor (Figure 4B).

#### 4.2.1 NRF2‐Linked Antioxidant Defense and Ferroptosis Suppression

Lactylation limits oxidative injury by reinforcing antioxidant and antiferroptosis programs through the NRF2 axis, iron homeostasis, and the GPX4/FSP1 defense system [189, 237, 238, 239, 240].

At the chromatin level, H3K18la‐mediated HECTD2 upregulation drives lenvatinib resistance through KEAP1 degradation and NRF2 activation, creating a lactate–lactylation–antioxidant reinforcing loop (Section 3.3.3). This histone‐based mechanism extends to iron‐regulatory loci: at the NFS1 promoter, H3K18la increases transcription and limits free iron accumulation, reducing ferroptotic pressure [241].

At the posttranscriptional level, IGF2BP3 K76 lactylation coordinates multiple ferroptosis‐suppression layers. In lenvatinib‐resistant HCC, it stabilizes PCK2 and NRF2 mRNAs, supporting metabolic rewiring and antioxidant defense [188]. In hepatic artery infusion chemotherapy‐resistant HCC, PARK7 acts as a lactyltransferase for IGF2BP3 K76, increasing its affinity for m^6^A‐modified FSP1 mRNA and promoting FSP1 expression [242]. This dual mechanism—stabilizing both NRF2 (for antioxidant transcription) and FSP1 (for lipid peroxide clearance)—illustrates how a single protein lactylation event can coordinate multiple layers of ferroptosis suppression and support drug resistance.

At the protein level, β‐catenin K193 lactylation enhances TCF4‐dependent GPX4 transcription in NSCLC, suppressing ferroptosis [239]. PRDX1 K67 lactylation promotes NRF2 nuclear translocation in HCC, inducing SLC7A11 (cysteine import), GPX4 (peroxide clearance), and HMOX1 (ROS reduction) [237]. Direct GPX4 lactylation has been reported mainly in noncancer oxidative injury models; its relevance to tumor therapy response remains unclear [243]. Current evidence supports indirect regulation of GPX4 and FSP1 through histone and RNA‐associated mechanisms rather than direct protein modification.

Collectively, these multilayer mechanisms establish a robust defense system: lactylation depletes proferroptotic iron while enhancing redox buffering capacity, enabling tumors to evade iron‐dependent cell death across diverse therapeutic contexts.

#### 4.2.2 NADPH Supply and Glutathione Metabolism

Protein lactylation also regulates the metabolic resources that determine how well tumor cells tolerate oxidative stress. Two systems are central here: NADPH production and glutathione synthesis.

For NADPH supply, KAT8‐mediated MDH2 K239 lactylation in renal cell carcinoma enhances MDH2 activity and its interaction with SLC25A1, promoting citrate export and supporting IDH1‐dependent NADPH generation [244, 245]. This reduces ROS accumulation and improves mitochondrial stress tolerance. The finding provides direct evidence that mitochondrial protein lactylation can support redox defense by increasing reducing‐equivalent supply. The PPP shows a more context‐dependent pattern. In cervical cancer, HPV16 E6 activates the pathway by inhibiting G6PD lactylation, implying that G6PD lactylation may restrain PPP activity in this setting [112, 246]. Noncancer studies further suggest that lactylation can affect G6PD and 6PGD, but their significance for therapy response in tumors remains uncertain [247].

For glutathione metabolism, lactylation reinforces the glutathione axis through multiple mechanisms. ACAT2‐catalyzed GCLM K34 lactylation increases glutamate‐cysteine ligase activity in KRAS‐mutant tumors, promoting glutathione production [248]. H4K12la accumulates at the GCLC promoter in colorectal cancer stem cells, activating GCLC transcription and contributing to oxaliplatin resistance [31]. NSUN2 K508 lactylation stabilizes GCLC mRNA in GC, further reinforcing glutathione production [158]. NAA20‐mediated ACF1 lactylation enhances GCLM‐dependent glutathione synthesis in neuroblastoma, supporting tumor progression [249]. These studies demonstrate that protein lactylation reinforces glutathione synthesis through direct enzyme modification, histone‐mediated transcriptional activation, RNA stabilization, and chromatin‐associated factor regulation.

Taken together, these mechanisms raise the oxidative damage threshold of tumor cells. This is especially relevant in treatment settings that depend on ROS accumulation, lipid peroxidation, or ferroptotic cell death.

#### 4.2.3 Bidirectional Control of Mitochondrial ROS and Ferroptosis

Protein lactylation does not always protect cells from oxidative injury. In some models, it increases ROS accumulation and enhances treatment‐induced death. This point is important because it argues against treating protein lactylation as a uniformly cytoprotective mark.

In prolactinoma, p300‐mediated H3K18la upregulates NDUFS7 and WASH1, leading to sustained mitochondrial ROS accumulation and increased sensitivity to dopamine receptor agonist‐induced apoptosis [250]. Here, histone lactylation amplifies rather than buffers oxidative stress because the downstream transcriptional output favors mitochondrial ROS production. The opposite pattern has been described in ferroptosis‐related settings. In lung adenocarcinoma, ferroptosis induction increases lactate production and triggers SUMO2 K11 lactylation. SUMO2 K11la disrupts ACSL4 SUMOylation homeostasis, promotes ACSL4 degradation, reduces lipid peroxidation, and suppresses ferroptosis [251]. In this case, lactylation acts as a feedback mechanism that limits oxidative membrane damage. Similarly, in melanoma resistant to BRAF/MEK inhibitors, LSD1 K503 lactylation facilitates its interaction with FosL1, preventing TRIM21‐mediated ubiquitination and degradation [238, 251, 252]. This process cooperatively downregulates TFRC transcription to reduce iron uptake and inhibit ferroptosis [238].

These findings illustrate a general principle: the redox consequence of lactylation is determined less by bulk lactate levels than by the identity of the modified substrate and the pathway it controls. For mechanistic interpretation, site‐specific lactylation events are therefore more informative than total lactate accumulation.

### 4.3 Protein Lactylation and Cell Cycle Regulation

Cell‐cycle control is a key determinant of therapy response. Many anticancer treatments depend on sustained checkpoint activation, mitotic arrest, or failure to complete division. Under these conditions, lactate accumulation and protein lactylation can reshape how tumor cells process proliferative stress [253, 254, 255]. The available evidence points to three main routes: attenuation of p53‐dependent checkpoint function, promotion of mitotic exit or slippage, and support of cyclin‐associated cell‐cycle progression. These mechanisms operate through both direct protein lactylation and lactate‐dependent signaling, together linking the lactate‐rich tumor state to reduced sensitivity to therapies that rely on durable arrest or apoptosis (Figure 4C).

#### 4.3.1 p53 Checkpoint Attenuation Through Dual Mechanisms

Among checkpoint regulators, p53 provides the clearest mechanistic link between protein lactylation and cell‐cycle control. p53 coordinates DNA damage signaling, cell‐cycle arrest, senescence, and apoptosis [17, 256]. When its activity is weakened, tumor cells are more likely to continue cycling despite therapeutic stress.

Direct p53 lactylation comes from AARS1‐mediated p53 lactylation. AARS1 senses lactate and catalyzes lactylation of p53 at K120 and K139 [17]. These modifications compete with acetylation, reduce p53 DNA‐binding capacity, impair liquid–liquid phase separation, and suppress p53‐dependent transcription [17]. Functionally, this weakens checkpoint enforcement and apoptotic output, especially in tumors that retain wild‐type p53. Indirect regulation through YY1 has been described through the YY1–FBXO33–p53 axis in gallbladder cancer. YY1 K183 lactylation enhances FBXO33 transcription, and FBXO33 in turn regulates p53 polyubiquitination at K291 and K292 [257, 258]. This pathway links protein lactylation to altered p53 turnover and reduced tumor‐suppressive control.

Collectively, these dual mechanisms—direct p53 modification and indirect YY1‐mediated regulation—converge to weaken p53 function. This results in reduced checkpoint stringency under treatment stress, enabling tumor cells to bypass cell‐cycle arrest and apoptosis.

#### 4.3.2 Lactate‐Dependent Mitotic Slippage

Cell‐cycle escape can also occur at mitosis. Although this mechanism is lactate‐dependent rather than a direct protein lactylation event, it is conceptually relevant to understanding how lactate‐rich environments facilitate therapy resistance.

The key example is remodeling of the anaphase‐promoting complex/cyclosome (APC/C). APC/C drives mitotic exit by promoting degradation of substrates such as cyclin B1 and securin. l‐Lactate directly inhibits the deSUMOylating enzyme SENP1, which stabilizes APC4 SUMOylation at K772 and K798, enhances APC/C binding to UBE2C, and promotes APC/C activation [255]. Activated APC/C accelerates cyclin B1 and securin degradation, thereby allowing cells to exit mitosis [259, 260, 261].

This pathway is particularly relevant to antimitotic therapy. Drugs that target mitosis aim to sustain spindle checkpoint activation and force prolonged arrest. Lactate‐driven APC/C activation can instead promote mitotic slippage, allowing cells to escape arrest before death is completed [255]. This lactate–APC/C axis illustrates how metabolic stress can bypass cell‐cycle checkpoints independently of protein lactylation, contributing to therapy resistance.

#### 4.3.3 Cyclin Lactylation and Proliferative Continuity

Beyond checkpoint failure and mitotic escape, lactylation can also support continued cell‐cycle progression. At the protein level, the strongest site‐specific evidence comes from cyclin E2 (CCNE2) in HCC. SIRT3 functions as a delactylase for CCNE2 and suppresses lactylation at K347 and K348, thereby limiting tumor growth [262, 263, 264]. This finding implies that CCNE2 lactylation favors G1/S progression and supports proliferative continuity under metabolic stress. In lung adenocarcinoma, the metabolic enzyme LDHC4 catalyzes RB1 lactylation at the K900 residue, which disrupts the RB1–E2F1 tumor‐suppressive complex, leading to cell cycle dysregulation and tumor progression [265].

At the transcriptional level, histone lactylation drives cell‐cycle gene expression. In lung cancer‐derived brain metastasis models, histone lactylation has been linked to increased CCNB1 transcription and accelerated DNA replication or cell‐cycle progression [266]. Similarly, protein lactylation upregulates CDK1 and CCND1 activity, accelerating G1/S transition and impairing radiation response [267, 268, 269, 270]. The current literature therefore supports a two‐tiered model: direct nonhistone lactylation (CCNE2, RB1) enhances cyclin activity and disrupts tumor suppressors, while histone lactylation activates cell‐cycle gene transcription (CCNB1, CDK1, CCND1). Together, these mechanisms enable tumor cells to maintain proliferative drive despite therapeutic pressure.

Taken together, Sections 4.1–4.3 define an early compensatory layer of tumor survival under therapeutic stress. By increasing repair capacity, shifting redox tolerance, and weakening cell‐cycle control, lactate‐linked signaling helps tumor cells withstand the initial injury imposed by treatment. This short‐term survival window may then support more durable forms of metabolic rewiring. The following sections therefore turn to longer‐term programs of tumor evolution, including malignant proliferation, metabolic reprogramming, TME remodeling, and cancer stem cell maintenance.

### 4.4 Protein Lactylation in Cell Proliferation and Malignant Transformation

The mechanisms discussed above explain how protein lactylation helps tumor cells survive early treatment stress. A separate but connected question is how these surviving cells maintain growth and acquire more stable malignant states. Cell proliferation and malignant progression depend on oncogenic transcription, growth signaling, metabolic feedback, and cellular plasticity. Protein lactylation connects these processes by converting lactate accumulation into chromatin‐level transcriptional changes or direct modification of signaling and metabolic proteins [85, 161, 262, 267, 268]. The available evidence supports a two‐tiered regulatory model: histone lactylation activating protumorigenic transcription, and nonhistone lactylation reinforcing proliferative signaling and cellular plasticity (Figure 5A).

**FIGURE 5:**
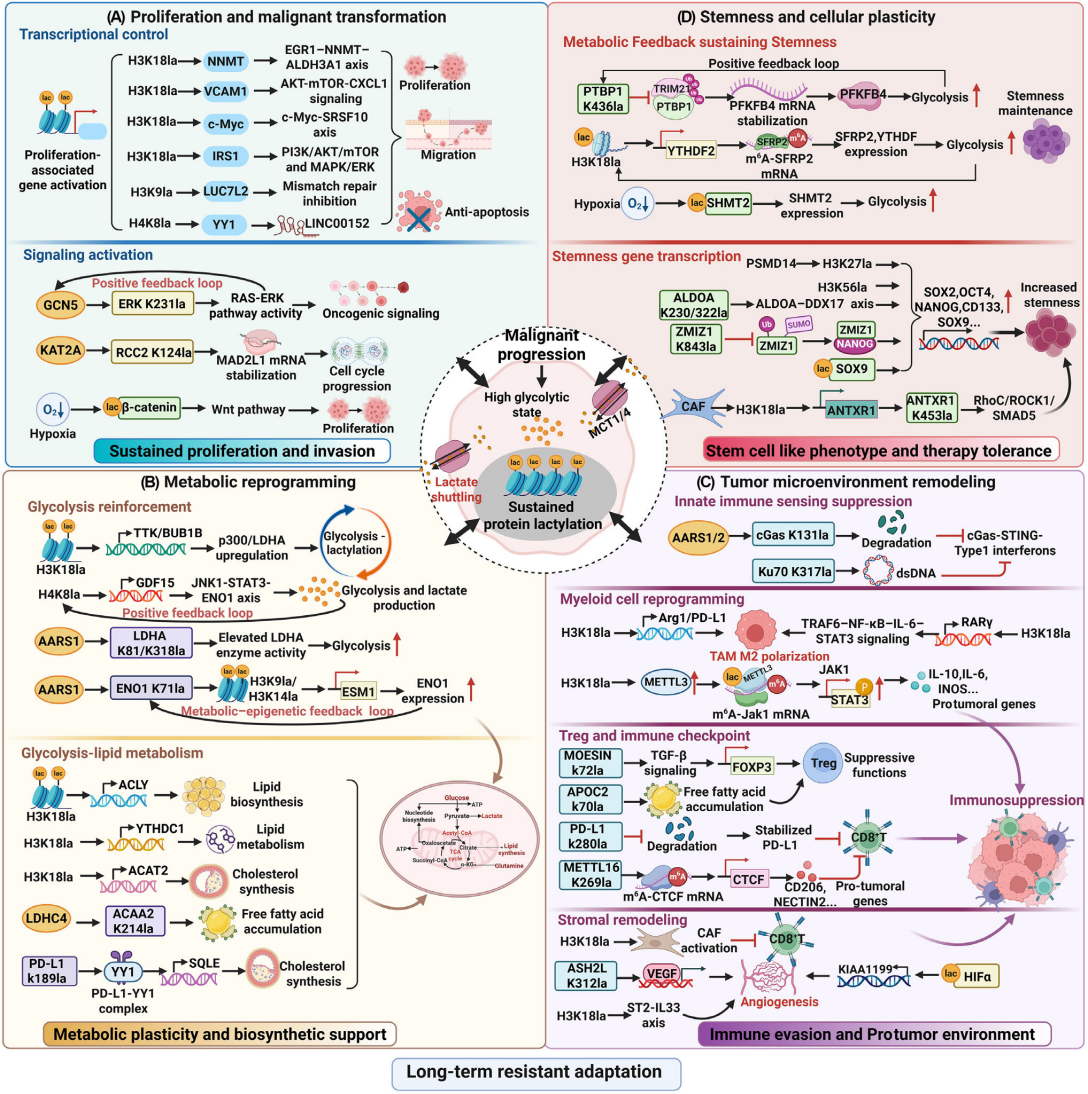
Lactylation‐driven malignant progression and microenvironment remodeling. Under sustained high glycolytic flux, tumor‐derived lactate drives persistent protein lactylation that extends beyond acute stress responses to coordinately reprogram transcription, metabolism, immune architecture, and stemness—establishing the long‐term basis for adaptive therapy resistance: (A) Proliferation and malignant transformation: H3K18la‐dependent activation of oncogenic transcription factors and positive‐feedback kinase signaling sustains tumor proliferation, migration, and apoptotic evasion. This metabolic‐to‐transcriptional coupling locks cells into a persistently malignant state under therapeutic pressure. (B) Metabolic reprogramming: Lactylation amplifies its own substrate supply by upregulating glycolytic enzymes while redirecting carbon flux toward lipid and cholesterol biosynthesis, forming a self‐reinforcing metabolic–epigenetic feedback loop. The resulting biosynthetic flexibility supports tumor growth under nutrient‐limited or drug‐imposed stress. (C) Tumor microenvironment remodeling: Lactylation suppresses innate immune sensing, polarizes macrophages toward an M2 phenotype, stabilizes PD‐L1, and activates CAFs and angiogenesis, collectively constructing an immunosuppressive protumor niche. This coordinated remodeling undermines both cytotoxic and immune checkpoint therapies. (D) Stemness and cellular plasticity: Lactylation sustains cancer stem cell identity by coupling glycolytic feedback loops to activation of core stemness transcription factors including SOX2, OCT4, and NANOG. This preserves a therapy‐tolerant stem‐like reservoir that serves as the cellular foundation for long‐term resistance. Abbreviations: iNOS, inducible nitric oxide synthase; NECTIN2, nectin cell adhesion molecule 2.

#### 4.4.1 Histone Lactylation Sustains Protumorigenic Transcription

Histone lactylation can activate genes involved in proliferation, invasion, epithelial–mesenchymal transition (EMT), angiogenesis, and therapy resistance. In HCC, elevated H3K9la and H3K56la are associated with poor prognosis. These marks activate ESM1 transcription and promote cell proliferation, migration, invasion, and EMT [11, 271, 272, 273].

Multiple tumor types demonstrate self‐reinforcing transcriptional circuits driven by histone lactylation. Aberrant EGR1 activation induces NNMT and ALDH3A1 expression, increasing lactate accumulation. Lactate then enhances EGR1 transcription through p300‐mediated H3K18 lactylation, maintaining the EGR1–NNMT–ALDH3A1 axis and supporting EGFR‐TKI resistance [187]. Consistent with the H3K18la‐driven autophagy program described in Section 3.3.4, histone lactylation also contributes to antiangiogenic therapy resistance in colorectal cancer through RUBCNL upregulation and autophagosome maturation [131]. In GC, H3K18la enrichment at the VCAM1 promoter promotes VCAM1 transcription, activating the AKT–mTOR–CXCL1 signaling axis and promoting GC cell proliferation and metastasis [274]. In HCC, PYCR1 upregulation promotes glycolysis and increases H3K18 lactylation. This increases IRS1 expression, activating the PI3K/AKT/mTOR and MAPK/ERK pathways and promoting tumor proliferation and metastasis [275].

Collectively, these findings demonstrate that histone lactylation can maintain transcriptional states that favor tumor growth, survival, and treatment resistance by coordinating transcriptional and signaling pathways to sustain malignant phenotypes under treatment pressure.

#### 4.4.2 Nonhistone Lactylation Reinforces Proliferative and Metabolic Signaling

Nonhistone lactylation can directly modify signaling proteins and metabolic enzymes, allowing lactate accumulation to influence tumor behavior beyond chromatin regulation. In glioblastoma, GCN5‐mediated ERK K231 lactylation activates the RAS–ERK pathway [205], Activated ERK further enhances GCN5 activity, forming a GCN5–ERK positive‐feedback loop, providing site‐specific evidence that lactylation can directly strengthen oncogenic signaling [205].

In TNBC, high LDHC4 expression promotes ACAA2 K214 lactylation [110]. This modification activates PI3K/AKT/mTOR‐associated autophagy and cell‐cycle programs, supporting tumor progression [110]. Similarly, in breast cancer, RCC2 K124 lactylation via KAT2A stabilizes MAD2L1 mRNA and drives cell cycle progression [276]. In lung adenocarcinoma, BZW2 promotes glycolysis and increases lactate production, facilitating IDH3G lactylation and tumor progression [277]. In colorectal cancer, hypoxia induces β‐catenin lactylation, which reinforces β‐catenin protein stability and expression, enhancing colorectal cancer cells survival and stem properties by activating the Wnt pathway [278].

These examples provide stronger mechanistic evidence because they define the modified substrate, lysine site, upstream regulator, and downstream pathway, supporting a substrate‐specific model of nonhistone lactylation [279]. The same lactate‐rich state can produce different biological outputs depending on whether protein lactylation modifies a kinase, metabolic enzyme, transcriptional regulator, or chromatin‐associated factor.

#### 4.4.3 Protein Lactylation Enhances Cellular Plasticity and Posttranscriptional Regulation

Malignant progression often requires cellular plasticity, including EMT, stemness acquisition, lineage transition, and compensatory responses to treatment pressure. Protein lactylation can support these states by changing chromatin accessibility and cooperating with RNA‐modification networks.

Protein lactylation coordinates transcriptional and posttranscriptional regulation through multiple mechanisms. In breast cancer, H3K18la enhances c‐Myc expression; c‐Myc then transcriptionally upregulates splicing factor SRSF10 to promote alternative splicing of MDM4 and Bcl‐x, thereby supporting proliferation and survival [161]. In glioblastoma, H3K9la mediates the retention of MLH1 intron 7 by activating LUC7L2 transcription, reducing MLH1 expression, inhibiting mismatch repair, and contributes to temozolomide resistance [231]. Protein lactylation also promotes stemness and cellular plasticity through noncoding RNA networks. In HCC, lactylome analysis has identified lactylation‐related mechanisms associated with liver cancer stemness [30]. In colorectal cancer, lactate‐induced H4K8la upregulates LINC00152 transcription by modifying its promoter, reducing the binding efficiency of transcription factor YY1. LINC00152 then promotes cell invasion and migration by inhibiting bacteria‐induced inflammation [280].

Together, these findings demonstrate that protein lactylation promotes cellular plasticity through a multilayer regulatory network: histone lactylation alters chromatin accessibility, while cooperation with alternative splicing and noncoding RNA pathways enables context‐dependent adaptation to treatment pressure.

### 4.5 Protein Lactylation Drives Metabolic Reprogramming in Tumor Cells

Metabolic reprogramming provides a substrate basis for protein lactylation, while protein lactylation can in turn reshape tumor metabolism. Current evidence suggests that protein lactylation reinforces glycolysis, links glucose metabolism to lipid synthesis, and directly regulates metabolic enzymes [32, 281, 282, 283, 284]. These mechanisms help tumor cells stabilize a high‐lactate state and redirect metabolic flux toward biosynthesis, stress tolerance, and sustained growth (Figure 5B). This establishes a bidirectional “metabolism–lactylation” feedback loop: enhanced glycolysis produces lactate that drives protein lactylation, while lactylation‐mediated transcriptional and enzymatic changes sustain glycolytic output. The metabolic consequence of protein lactylation depends on the modified substrate, lysine site, tumor context, and position of the target protein within the metabolic network.

#### 4.5.1 Histone Lactylation Reinforces Glycolytic Programs

Histone lactylation can maintain glycolytic activity through transcriptional feedback, forming self‐reinforcing metabolic–epigenetic circuits. In pancreatic ductal adenocarcinoma, enhanced glycolysis increases lactate accumulation and H3K18la. H3K18la activates TTK and BUB1B transcription, while TTK and BUB1B further upregulate p300 and LDHA, sustaining glycolysis and protein lactylation [115]. This glycolysis–H3K18la–TTK/BUB1B circuit shows how histone lactylation can preserve a high‐lactate metabolic state. Similarly, in colon epithelial cells, GDF15 upregulates ENO1 through the JNK1–STAT3 pathway, increasing glycolysis and lactate production. Lactate then promotes GDF15 transcription through H4K8la, forming a GDF15–ENO1–lactate–H4K8la loop [285]. In ovarian cancer, AARS1 mediates ENO1 K71 lactylation. This modification increases lactate synthesis and promotes H3K9la and H3K14la. These histone marks upregulate ESM1 transcription and support ENO1 expression, forming a metabolic–epigenetic feedback loop. Secreted ESM1 also enhances endothelial cell survival and angiogenesis, contributing to bevacizumab resistance [193].

Collectively, these self‐reinforcing circuits demonstrate that histone lactylation acts as a transcriptional stabilizer of glycolysis, not simply reflecting lactate accumulation, but actively maintaining the metabolic program that produces lactate.

#### 4.5.2 Protein Lactylation Links Glycolysis With Lipid Metabolism

Lactylation can also extend glycolytic reprogramming toward lipid metabolism. This is important because rapidly growing tumor cells require not only ATP and glycolytic intermediates, but also lipid precursors for membrane synthesis and signaling.

At the transcriptional level, histone lactylation activates lipogenic gene expression. In HCC, H3K18la promotes YTHDC1 transcription and remodels lipid metabolism [286]. In pancreatic cancer, PSMD14 stabilizes LDHA and increases lactate production. Elevated lactate promotes H3K18la, which upregulates ACLY transcription and enhances lipid synthesis [287]. Since ACLY converts citrate‐derived carbon into acetyl‐CoA, this pathway connects glycolysis‐derived lactate with acetyl‐CoA supply and lipid biosynthesis. This lactate–ACLY axis illustrates how protein lactylation bridges glycolytic carbon flux with de novo lipogenesis. Beyond lipid synthesis, in pancreatic cancer, H3K18la activates ACAT2 transcription, boosting cholesterol synthesis. Tumor‐derived cholesterol is then transferred to tumor‐associated macrophages via small extracellular vesicles, reinforcing their immunosuppressive M2 polarization [288, 289, 290]. At the protein level, nonhistone lactylation directly modulates lipid metabolic enzymes. In TNBC, high LDHC4 expression promotes ACAA2 K214 lactylation. This modification increases free fatty acid accumulation and supports tumor progression [110].

The metabolic consequence of lactylation depends on where the modified enzyme sits in the pathway. In some cases, lactylation increases metabolic output. In others, it may restrain a specific enzymatic step, alter pathway balance, or redistribute metabolic flux. Site‐resolved functional studies are therefore essential for distinguishing causal metabolic regulation from broader lactylome association.

### 4.6 Protein Lactylation Remodels the TME

The TME is shaped by lactate accumulation, immune suppression, stromal activation, and abnormal vasculature. Protein lactylation connects these metabolic and cellular changes by modifying histones and nonhistone proteins in tumor, immune, and stromal cells (Figure 5C). The available evidence demonstrates that protein lactylation orchestrates TME remodeling through four interconnected mechanisms: suppressing innate immune sensing, reprogramming immunosuppressive cell states, remodeling stromal compartments, and driving angiogenic signaling [18, 34, 217, 291, 292].

#### 4.6.1 Innate Immune Sensing Suppression

DNA‐damaging therapies, including radiotherapy and chemotherapy, can generate micronuclei and cytosolic double‐stranded DNA. Upon micronuclear rupture, cGAS recognizes the exposed DNA, catalyzes cGAMP production, thereby activating STING‐dependent Type I interferon signaling and linking treatment‐induced genomic damage to antitumor immunity [293, 294].

Protein lactylation can suppress this DNA‐sensing pathway through two complementary mechanisms. In glioblastoma, tumor‐associated macrophages export lactate through MCT4, while glioblastoma stem‐like cells take up lactate through MCT1. This intercellular lactate transfer induces Ku70 lactylation at K317, facilitating Ku70–Ku80–DNA–PKcs complex formation and enhancing NHEJ‐mediated DNA repair. As a result, it reduces the persistence and cytosolic accumulation of damaged DNA, thereby suppressing cGAS–STING–interferon signaling. Blocking lactate transport or Ku70 lactylation restores cGAS–STING activity and improves immune checkpoint blockade in glioblastoma models [221]. Additionally, intracellular l‐lactate can be sensed and catalyze the lactylation of cGAS to inactivate cGAS. AARS1/2‐mediated cGAS K131 lactylation reduces the DNA‐binding capacity of cGAS and inhibits cGAMP production, thereby suppressing the innate immune surveillance [18]. Collectively, these mechanisms connect lactate metabolism with DNA repair and innate immune evasion across treatment settings: protein lactylation reduces the DNA signal available for cGAS activation by enhancing DNA repair and directly weakens the DNA‐sensing function of cGAS itself.

#### 4.6.2 Immunosuppressive Myeloid Cell Reprogramming

Myeloid cells provide some of the clearest evidence for lactylation‐dependent immune suppression in the TME. Protein lactylation can shift macrophages and tumor‐infiltrating myeloid cells toward anti‐inflammatory or tumor‐supportive states through coordinated histone and nonhistone modifications, thereby establishing an immune context that favors tumor persistence and reduced therapeutic responsiveness.

Macrophage M2 polarization is driven by histone lactylation across multiple tumor types. In HCC, tumor‐cell PFKFB4 promotes glycolysis and lactate production. Lactate increases H3K18la in tumor‐associated macrophages, where H3K18la is enriched at the promoters of Arg1 and CD206, promoting M2‐like polarization [220]. In glioblastoma, glucose‐driven histone lactylation in monocyte‐derived macrophages has been linked to IL‐10 production and immunosuppressive activity [291]. In colorectal cancer, macrophage H3K18la suppresses RARγ transcription, unleashing the TRAF6–NF‐κB–IL‐6–STAT3 signaling axis and further reinforcing M2 polarization [26].

Protein lactylation also cooperates with RNA m^6^A modification to strengthen immunosuppressive programming [58]. In colon cancer, lactate induces METTL3 upregulation through H3K18 lactylation. METTL3 modifies Jak1 mRNA, and the m^6^A–YTHDF1 axis enhances JAK1 translation and STAT3 phosphorylation. METTL3 lactylation at K281/K345 further supports its target RNA capture, strengthening immunosuppressive myeloid‐cell function [58]. This example shows how histone lactylation, nonhistone lactylation, and RNA modification can converge on the same suppressive immune program.

Beyond macrophages, neutrophil immunosuppression is also lactylation dependent. In brain tumor‐infiltrating neutrophils, hypoxia drives enhanced glucose metabolism in the CD71^+^ subset, leading to high lactate production. This lactate induces histone lactylation, which regulates Arg1 expression and endows CD71^+^ neutrophils with immunosuppressive properties [295].

#### 4.6.3 Treg and Checkpoint‐Associated Immune Suppression

Regulatory T cells can tolerate lactate‐rich conditions through lactylation‐dependent signaling that enhances their suppressive capacity and metabolic fitness. MOESIN K72 lactylation strengthens TGF‐β‐related signaling and supports FOXP3‐associated Treg suppressive function under metabolic stress [217]. In glioblastoma, H3K18la‐dependent CCR8 upregulation in Tregs disrupts the Th17/Treg balance, facilitating immune evasion [296]. In malignant pleural effusion, protein lactylation activates the NF‐κB p65/TNFR2 axis, enhancing Treg suppression and CD8^+^ T‐cell inhibition [297]. In NSCLC, APOC2 K70 lactylation triggers free fatty acid release to promote Treg accumulation and anti‐PD‐1 resistance [218]. In diabetes‐associated pancreatic ductal adenocarcinoma, lactate enters tumor‐associated Schwann cells through MCT1/MCT4 and induces METTL16 K269 lactylation. This modification enhances m^6^A‐dependent CTCF stability, activates immunosuppressive ligand transcription, impairs CD8^+^ T‐cell function, and promotes resistance to PD‐1 blockade [298].

Protein lactylation also regulates immune checkpoint pathways through site‐specific PD‐L1 modifications with distinct functional outcomes. Recent studies have identified multiple lactyltransferases and lactylation sites on PD‐L1, establishing a multilayer regulatory network that controls PD‐L1 stability, localization, and function. HAT1 catalyzes PD‐L1 lactylation at K75 and K178 within the extracellular domain [299]. This lactylation‐dependent modification protects PD‐L1 from ER‐associated degradation and promotes ER‐to‐Golgi trafficking, thereby stabilizing PD‐L1 protein levels and tumor immune evasion [299]. AARS1‐mediated PD‐L1 K280 lactylation blocks HUWE1‐dependent ubiquitination and degradation, stabilizing PD‐L1 and enhancing suppression of CD8^+^ T‐cell activity [51]. In HCC, PD‐L1 K189 lactylation has a different function: it promotes PD‐L1 binding to VIM and facilitates nuclear translocation, where nuclear PD‐L1 cooperates with YY1 to activate SQLE transcription, enhancing cholesterol synthesis and supporting tumor growth [209]. These findings show that PD‐L1 lactylation can act through more than one mechanism. It may stabilize membrane PD‐L1 and strengthen immune checkpoint signaling, or it may promote nuclear PD‐L1 functions that support tumor metabolism and growth. The biological outcome therefore depends on the modified lysine site and tumor context.

#### 4.6.4 Stromal and Vascular Compartment Reprogramming

Lactylation‐dependent remodeling also occurs in nonimmune stromal cells, altering stromal barrier formation, and vascular support. In CAFs, tumor‐derived lactate drives fibrotic remodeling. In PDAC, tumor glycolysis and lactate accumulation increase H3K18la in CAFs. Elevated H3K18la promotes CAF activation and extracellular matrix deposition, contributing to a fibrotic microenvironment that can restrict CD8^+^ T‐cell infiltration [300].

In the vascular compartment, protein lactylation regulates angiogenesis through complementary tumor‐cell‐intrinsic and endothelial‐cell‐associated mechanisms. In HCC, ASH2L K312 lactylation promotes chromatin opening at the VEGFA promoter and enhances VEGFA transcription, thereby supporting angiogenesis [301]. In prostate cancer, lactylated HIF1α has been reported to increase KIAA1199 expression, promoting VEGFA release, angiogenesis, and vasculogenic mimicry [22]. In melanoma endothelial cells, H3K18la upregulates ST2, increases responsiveness to IL‐33, and activates Akt‐mediated angiogenesis [272].

Collectively, these studies demonstrate that protein lactylation remodels the TME through cell‐type‐ and context‐dependent regulation of immune, stromal, and vascular processes, including immunosuppressive cell programming, checkpoint regulation, stromal barrier formation, and abnormal angiogenesis. Rather than a passive consequence of lactate accumulation, protein lactylation functions as an active regulatory network within the TME. By promoting immune‐excluded or immune‐tolerant states, protein lactylation may weaken innate and adaptive antitumor immunity and reinforce stromal or vascular barriers, thereby reducing responses to immunotherapy and microenvironment‐directed treatments [18, 218, 221, 291, 297]. This establishes protein lactylation as a central orchestrator of TME remodeling, integrating metabolic stress with immune evasion, stromal barriers, and vascular support to enable tumor progression and therapy resistance. However, its therapeutic relevance likely varies across tumor contexts, and further studies are needed to define the key cell types, substrates, enzymes, spatial settings, and treatment conditions involved.

### 4.7 Protein Lactylation and Cancer Stem Cells

Cancer stem cells (CSCs) are tumor subpopulations with self‐renewal capacity, differentiation potential, and high tolerance to therapeutic stress. They contribute to tumor recurrence, metastasis, and treatment resistance. Current evidence suggests that protein lactylation links lactate accumulation to CSC maintenance by regulating metabolic feedback, stemness‐associated transcription, and therapy‐tolerant survival programs [30, 31, 101, 302]. Rather than passively reflecting lactate accumulation, protein lactylation actively enforces a CSC‐like state to preserve metabolic flexibility, transcriptional plasticity, and stress tolerance, suggesting that the lactylation–stemness axis may contribute to treatment resistance across multiple tumor contexts (Figure 5D).

#### 4.7.1 Metabolic Feedback Sustaining CSC Traits

CSC‐like cells often survive in nutrient‐ and oxygen‐variable environments through flexible metabolic programs. Protein lactylation can reinforce this state by stabilizing glycolytic feedback circuitsthat form self‐perpetuating “lactate–glycolysis–lactylation” loops. In glioma stem cells, lactate induces PTBP1 K436 lactylation, which enhances PTBP1 binding to the PFKFB4 mRNA 3′UTR and increases PFKFB4 expression. This lactate–PTBP1–PFKFB4 loop sustains glycolytic flux and supports glioma stemness [303]. Similarly, in NSCLC, elevated H3K18 lactylation enriches at the distal promoter region of YTHDF2, promoting its transcriptional expression. YTHDF2 recognizes m^6^A modification sites on SFRP2 mRNA, reducing its stability and thereby suppressing SFRP2 expression. Downregulation of SFRP2 activates the Wnt/β‐catenin pathway, promoting glycolysis and cancer stem cell properties [102]. In esophageal squamous cell carcinoma, hypoxia induces SHMT2 lactylation, which upregulates SHMT2 protein expression and subsequently facilitates glycolysis and stemness maintenance [304]. Collectively, these self‐reinforcing metabolic circuits demonstrate that lactylation maintains CSC traits through both tumor‐cell‐intrinsic glycolytic feedback and hypoxia‐induced metabolic reprogramming, linking metabolic stress to CSC survival and therapy evasion.

#### 4.7.2 Stemness‐Associated Transcriptional Regulation

Protein lactylation can also support CSC identity by altering chromatin accessibility and transcriptional regulator function through coordinated histone and nonhistone modifications. At the chromatin level, histone lactylation establishes accessibility at pluripotency loci. In liver cancer stem cells, glycolysis‐derived lactate increases H3K56la and ALDOA K230/K322 lactylation. H3K56la enhances chromatin accessibility at pluripotency‐related loci, including OCT4, SOX2, and NANOG, while ALDOA lactylation induces DDX17 nuclear translocation to synchronize transcriptional and RNA processing programs, thereby supporting liver CSC stemness [30]. In gastric adenocarcinoma, highly expressed PSMD14 strengthens tumor stemness and drives tumor progression by increasing glycolysis and lactate accumulation, which activates H3K27la and turns to enhance the expression of PSMD14 and SOX9 [305].

At the protein level, nonhistone lactylation stabilizes transcriptional regulators and enhances their stemness‐promoting activity. In breast cancer, lactate induces ZMIZ1 K843 lactylation. This modification suppresses SUMOylation and ubiquitination, stabilizes ZMIZ1, and increases ZMIZ1 binding to Nanog. Enhanced Nanog transcriptional activity activates downstream targets such as OCT4 and NPC2, promoting stemness, cholesterol accumulation, and tamoxifen resistance [306]. Similarly, in NSCLC, hypoxia‐induced SOX9 lactylation has been linked to increased NANOG and CD133 expression [281]. Together, these studies show that protein lactylation can regulate CSC programs through histone marks, metabolic enzymes, and transcriptional coregulators.

#### 4.7.3 CSC‐Related Therapy Resistance

CSCs survive treatment by maintaining stemness and activating stress–tolerance pathways. Protein lactylation contributes to this process by linking stemness regulation with redox control, metabolic reprogramming, and drug resistance through multiple interconnected mechanisms. In colorectal cancer stem cells, the p300–H4K12la–GCLC axis promotes glutathione synthesis and antioxidant defense, increasing chemotherapy resistance [31]. This mechanism connects histone lactylation with redox protection under cytotoxic stress. Additionally, stromal‐derived lactate can enhance CSC properties and therapy resistance. The CAF‐derived lactate–ANTXR1 axis stabilizes ANTXR1 and activates the RhoC/ROCK1/SMAD5 signaling cascade to sustain stemness and confer oxaliplatin resistance [125]. Similarly, the ZMIZ1 lactylation pathway promotes CSC‐like traits, cholesterol accumulation, and tamoxifen resistance in breast cancer [306]. These mechanisms reveal that protein lactylation supports therapy‐resistant CSC states through coordinated control of stemness programs, metabolic flexibility, and stress tolerance pathways. This establishes protein lactylation as a central enforcer of CSC phenotypes that underpin treatment failure, tumor recurrence, and metastasis.

Together, Section 4 highlights protein lactylation as a context‐dependent regulatory layer that connects lactate metabolism with therapy resistance. By modulating DNA repair, oxidative stress control, cell‐cycle regulation, malignant proliferation, metabolic reprogramming, TME remodeling, and cancer stem cell maintenance, protein lactylation can support both acute survival and longer‐term resistant states [13, 31, 109, 221, 300]. These findings provide a mechanistic basis for considering the lactate–lactylation axis as a therapeutic vulnerability. The next section therefore focuses on intervention strategies, including metabolic targeting, regulation of protein lactylation writers and erasers, substrate‐specific approaches, and rational combination therapies.

## 5 Therapeutic Targeting of Protein Lactylation: From Preclinical Evidence to Clinical Trials

Protein lactylation is increasingly recognized as a therapeutically relevant node linking tumor metabolism to therapy resistance, immune evasion, and adaptive stress responses. Current interventions mainly act at four levels: lactate production, lactate transport, lactylation‐modifying enzymes, and key lactylated substrates. These strategies form a hierarchical targeting framework, progressing from broad metabolic disruption to site‐specific inhibition. However, most available agents still operate at the lactate supply level, with site‐specific lactylation inhibitors remaining scarce but representing the most promising direction for precision targeting (Figure 6).

**FIGURE 6:**
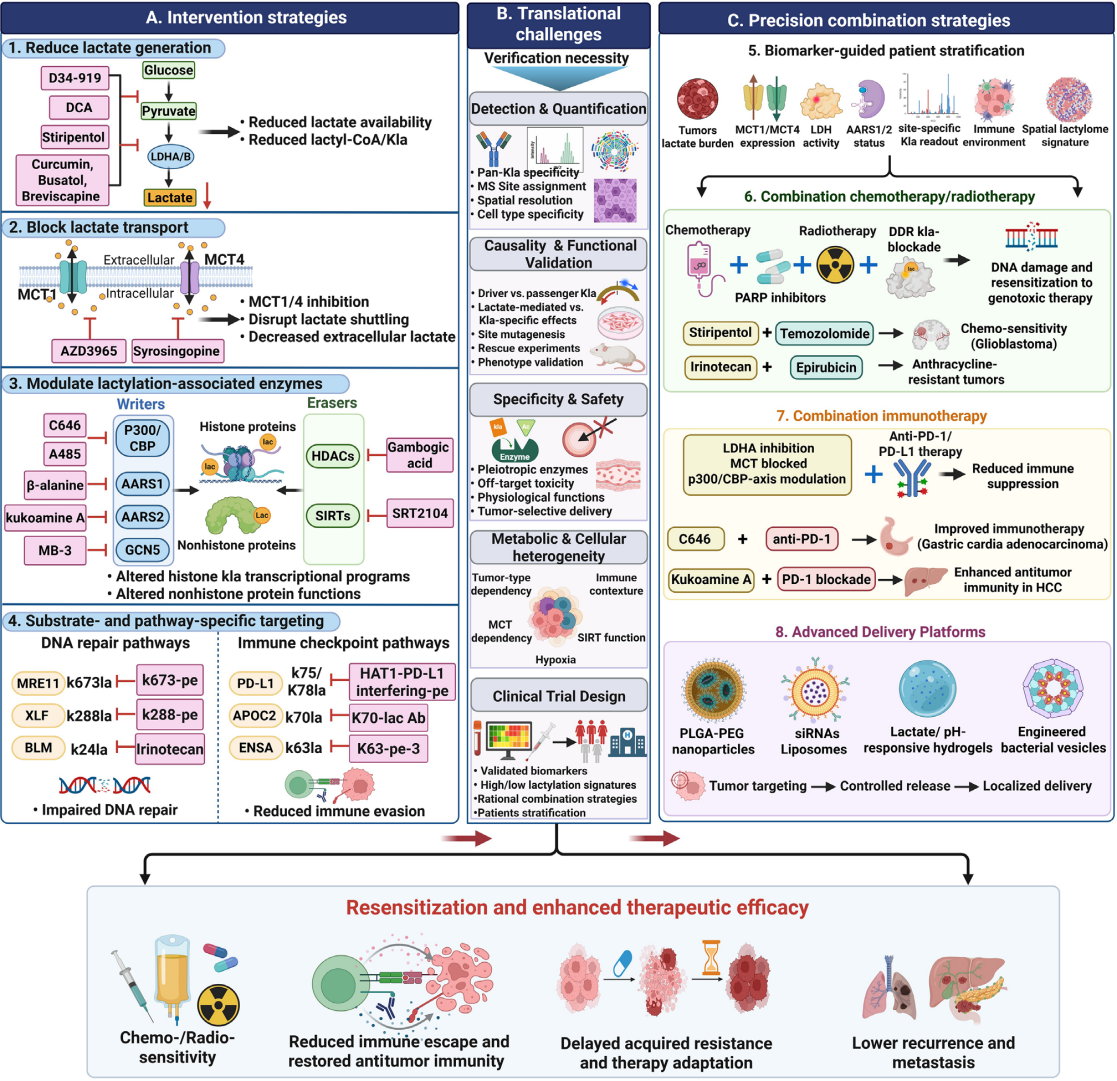
Preclinical intervention strategies and translational roadmap targeting the protein lactylation. Multilevel targeting of the lactate–lactylation axis, combined with biomarker‐guided stratification, enables rational regimens that resensitize tumors to chemotherapy, radiotherapy, and immunotherapy. (A) Intervention strategies: Four hierarchical intervention points: (1) reducing lactate generation; (2) blocking lactate transport; (3) modulating writers or erasers; (4) substrate‐specific targeting DNA repair and immune evasion. (B) Translational challenges: Clinical translation requires robust detection tools, causality validation distinguishing driver from passenger modifications, specificity to minimize off‐target toxicity, and strategies addressing metabolic and cellular heterogeneity across tumor types and immune contexts. (C) Precision combination strategies: Biomarker‐guided stratification identifies responsive patients. (5–6) Combination chemo‐/radiotherapy pairs genotoxic agents with DDR Kla‐blockers. (7) Combination immunotherapy cotargets lactylation‐driven suppression. (8) Advanced delivery platforms (nanoparticles, liposomes, pH‐responsive hydrogels, bacterial vesicles) enable tumor‐targeted, controlled release. These precision frameworks restore sensitivity, reduce immune suppression, delay resistance, and lower recurrence. Abbreviation: MS, mass spectrometry.

### 5.1 Reducing Lactate Production

Reducing lactate supply is the most direct upstream strategy. By limiting lactate availability, this approach indirectly decreases protein lactylation, including modifications on both histone and nonhistone proteins involved in treatment resistance (Figure 6A).

LDHA inhibition reverses lactylation‐driven therapy resistance across multiple tumor types. In glioblastoma, stiripentol, a United States Food and Drug Administration (US FDA)‐approved antiepileptic drug with LDHA inhibitory properties, inhibits LDHA/B, lowers H3K9la, restores MLH1 expression through the LUC7L2/MLH1 axis, and resensitizes tumors to temozolomide both in vitro and in vivo [231]. Stiripentol also reduces NBS1 K388 lactylation, impairs HR repair, and overcomes chemotherapy resistance in GC models [39]. Collectively, these site‐specific effects demonstrate that LDHA inhibition can reverse lactylation‐driven DNA repair enhancement and restore treatment sensitivityby depleting the lactate substrate pool.

Beyond LDHA, targeting upstream glycolytic enzymes and metabolic pathways also reduces lactylation. D34‐919 suppresses glycolytic flux by disrupting the ALDH1A3–PKM2 interaction and enhances sensitivity to chemoradiotherapy in glioblastoma [150]. In HCC, dichloroacetate, a pyruvate dehydrogenase kinase inhibitor, promotes oxidative metabolism of pyruvate rather than its conversion to lactate, thereby reducing lactate accumulation and lactylation [307, 308]. Additionally, natural compounds such as curcumin, busatol, and breviscapine have been associated with reduced lactylation‐related phenotypes and improved antitumor effects, though their mechanisms remain less precisely defined [309, 310, 311, 312].

These findings establish lactate restriction as a practical entry point for disrupting the protein lactylation network. However, most effects remain lactylation‐associated rather than lactylation‐specific, as global lactate depletion affects multiple downstream pathways beyond protein lactylation. Systemic inhibition of glycolysis may induce adverse effects such as gastrointestinal disorders and mediastinal disorders and may compromise antitumor immunity, which is also glycolysis‐ and lactylation dependent [313, 314, 315, 316]. These limitations underscore the need for more selective targeting strategies that disrupt oncogenic lactylation without impairing protective immune functions.

### 5.2 Blocking Lactate Transport

Blocking lactate transport can interrupt lactate exchange between tumor cells and the microenvironment, thereby disrupting both tumor‐intrinsic lactylation and lactate‐driven immunosuppression. This approach mainly targets MCT family members, especially MCT1 and MCT4, which mediate bidirectional lactate flux across cellular membranes.

MCT1 inhibition demonstrates dual benefits: reversing tumor‐intrinsic lactylation and restoring immune surveillance. AZD3965, a selective MCT1 inhibitor currently in Phase I clinical trials, enhances radiosensitivity and disrupts stress‐induced responses to DNA damage [132, 166, 317, 318]. MCT1 blockade also reduces cGAS K131 lactylation and restores innate immune surveillance in stressed mice [18]. In malignant pleural effusion models, AZD3965 combined with anti‐PD‐1 treatment significantly prolonged survival [297]. Syrosingopine has likewise been linked to altered nonhistone lactylation, impaired DNA repair, and improved radiotherapy response [319]. Together, these findings demonstrate that MCT inhibition can simultaneously disrupt tumor‐intrinsic lactylation and restore immune surveillance.

However, MCT‐targeted therapy is highly context‐dependent. Transporter expression, lactate flux, and metabolic compensation differ across tumor types [37, 320, 321]. In some tumors, MCT4 upregulation may compensate for MCT1 inhibition, limiting therapeutic efficacy. Dual MCT1/MCT4 inhibition or combination with other metabolic inhibitors may be required in these contexts to achieve durable lactate depletion and overcome adaptive resistance.

### 5.3 Targeting Lactylation‐Modifying Enzymes

More proximal intervention can be achieved by targeting lactylation writers or erasers. While lactylation readers—proteins that recognize and interpret lactyl‐lysine marks—represent an emerging therapeutic frontier, current translational efforts focus primarily on writers and erasers, for which validated inhibitors already exist and demonstrate preclinical efficacy. Recent studies have identified Brg1, DPF2, and TRIM33 as the first lactylation readers, which recognize histone lactylation through their bromodomains and PHD fingers to modulate chromatin accessibility and transcription [68, 69, 86]. Although reader‐targeted therapies—analogous to bromodomain inhibitors used against acetylation readers—hold promise for disrupting lactylation‐dependent transcriptional programs, such agents remain in early development. Current therapeutic strategies therefore concentrate on writers and erasers, where molecular tools are more mature.

#### 5.3.1 Writer Inhibition

Inhibiting lactyltransferases disrupts protein lactylation at its source, offering a more targeted alternative to global lactate depletion. p300/CBP inhibitors demonstrate broad efficacy across tumor types. C646 reduces H3K18la at the PDCD1 promoter, reverses CD8^+^ T‐cell exhaustion, and improves anti‐PD‐1 efficacy in gastric cardia adenocarcinoma models [216]. A485 enhances radiosensitivity in gliomas [148, 322]. while C646 combined with oxamate suppresses CREB lactylation in vivo [323]. These findings establish p300/CBP as tractable therapeutic targets, though their pleiotropic effects on multiple acylations necessitate careful selectivity assessment.

Beyond p300/CBP, AARS1 and AARS2 have been identified as global lysine lactyltransferases and intracellular l‐lactate sensors [18]. These lactyl‐CoA‐independent enzymes offer distinct advantages: they selectively transfer lactyl groups without affecting other acylations, and they integrate lactate sensing with substrate selection. AARS2‐mediated cGAS lactylation suppresses innate immune sensing, while AARS2‐dependent AP‐2γ K444 lactylation promotes HCC progression. This axis can be disrupted by kukoamine A nanotherapy, which also synergizes with PD‐1 blockade [18]. β‐Alanine, a common sports supplement, has been identified as an AARS1 inhibitor. In animal models, β‐alanine hinders lactate binding to AARS1, prevents Kla‐dependent inactivation of tumor suppressor p53, at K120 and K139, and reduces tumorigenesis [17, 324]. β‐Alanine's dual safety profile—widely used as an ergogenic aid with minimal side effects—and its selective disruption of protein lactylation without impairing protein translation make it an attractive lead compound for clinical translation.

Other KAT family members also mediate oncogenic protein lactylation. MB‐3, a GCN5 inhibitor, ameliorates cisplatin resistance in ovarian cancer [119]. In glioblastoma, GCN5‐mediated ERK K231 lactylation activates the RAS–ERK pathway, and GCN5 inhibition disrupts this positive‐feedback loop [205]. Collectively, these writer‐targeted strategies demonstrate that disrupting lactyltransferases can reverse therapy resistance, restore immune surveillance, and disrupt oncogenic signaling—though substrate promiscuity remains a challenge for broad‐spectrum KAT inhibitors.

#### 5.3.2 Eraser Modulation

By contrast, eraser‐directed strategies remain harder to generalize because HDAC‐ and SIRT‐mediated delactylation is strongly substrate and context dependent [10]. Unlike writers, which universally install lactyl marks, erasers exhibit dual and sometimes opposing roles: they can remove oncogenic protein lactylation to suppress tumorigenesis, or they can remove protective protein lactylation to enable immune evasion. This substrate‐dependent duality complicates therapeutic application.

In contexts where protein lactylation drives oncogenic programs, eraser inhibition enhances protein lactylation‐dependent antitumor effects. Gambogic acid [325] and SRT2104 (a SIRT1 inhibitor) [326] disrupt lactylation‐positive feedback loops by inhibiting delactylation, thereby stabilizing histone lactylation that antagonizes oncogenic transcription and achieving tumoricidal and therapy‐sensitizing effects. However, SIRT3 also functions as a lactylation eraser that removes immunosuppressive lactyl marks to suppress immune evasion in breast cancer cells and enhance PD‐1 blockade efficacy in melanoma models [10, 262, 327]. In these contexts, SIRT3 activation—rather than inhibition—produces therapeutic benefit. This paradox underscores a critical challenge: eraser modulation must be guided by substrate‐specific profiling rather than blanket activation or inhibition. Future strategies may require selective eraser inhibitors that target specific lactylation sites, or biomarker‐driven patient stratification to predict whether eraser activation or inhibition will produce benefit in a given tumor context.

### 5.4 Substrate‐Level Intervention: Targeting Key Lactylation Axes

Targeting defined lactylated substrates offers the highest conceptual specificity. This approach avoids the systemic effects of metabolic or enzyme‐wide inhibition by selectively blocking protein lactylation at a particular lysine residue on a defined protein. Site‐specific interventions represent the apex of the hierarchical targeting framework introduced in Section 5, offering precision that upstream strategies cannot achieve. By disrupting individual protein lactylation events that drive specific resistance mechanisms—such as DNA repair proficiency or immune checkpoint stabilization—these approaches promise maximal therapeutic selectivity with minimal collateral damage to normal cellular functions.

#### 5.4.1 DNA Repair Protein Lactylation

Lactylation of DNA repair proteins such as MRE11 and NBS1 enhances HR and contributes to resistance to DNA‐damaging therapy [13, 39]. Cell‐penetrating peptides designed to mimic protein lactylation motifs enable site‐specific blockade. K673‐pe, a cell‐penetrating peptide designed to mimic the sequence around the lactylation site K673 of MRE11, reduces MRE11 K673 lactylation, blocks DNA end resection, and impairs HR repair in response to cisplatin, thereby increasing chemosensitivity [13]. Similarly, XLF K288‐blocking peptide specifically inhibits XLF lactylation, impairs NHEJ repair, and sensitizes tumors to chemotherapy. Beyond synthetic peptides, some US FDA‐approved drugs disrupt protein lactylation through serendipitous mechanisms. Irinotecan, a Topoisomerase I inhibitor has been reported to suppress BLM K24 lactylation, inhibit HR repair, and alleviate chemoresistance in bladder cancer [13]. These findings suggest that some existing chemotherapeutic agents may exert part of their efficacy by disrupting specific protein lactylation events, opening opportunities for drug repurposing guided by protein lactylation profiling.

Collectively, these DNA repair‐targeted interventions demonstrate that site‐specific lactylation blockade can selectively disable therapy‐resistance mechanisms without broadly impairing genome maintenance in normal tissues—a critical advantage over pan‐HDAC or pan‐KAT inhibitors.

#### 5.4.2 Immune Checkpoint Protein Lactylation

Site‐specific lactylation blockade also extends to the immune regulatory network where protein lactylation stabilizes checkpoint proteins and drives immunosuppression. Interfering peptides and function‐blocking antibodies offer complementary approaches to dismantle these lactylation‐dependent immune barriers.

HAT1 functions as a lactyltransferase catalyzing PD‐L1 lactylation at K75 and K178, which stabilizes PD‐L1 through ER‐to‐Golgi trafficking and promotes tumor immune evasion. Mutation of these protein lactylation sites or treatment with HAT1‐PD‐L1 interfering peptides inhibit tumor progression and potentiate anti‐PD‐1 immunotherapy [299]. This strategy directly targets the glycosylation‐dependent stabilization mechanism identified in Section 4.6.3, offering a rational alternative to pan‐HAT1 inhibition.

Function‐blocking antibodies provide an orthogonal approach by recognizing the lactylated epitope itself. Anti‐APOC2 K70la monoclonal antibody (K70‐lac Ab) specifically blocks APOC2 K70 lactylation, reduces Treg accumulation, and overcomes anti‐PD‐1 resistance in NSCLC [218]. Similarly, ENSA K63‐blocking peptide (K63‐pe‐3) disrupts ENSA lactylation and downstream CCL2/CCR2 signaling, enhancing the efficacy of immune checkpoint blockade in PDAC preclinical models [152, 328]. These immune checkpoint‐targeted strategies establish that individual Kla events can directly control both tumor‐intrinsic and immune‐related phenotypes. Site‐specific interventions offer the potential for highly tumor‐selective lactylation inhibition with fewer off‐target effects than upstream metabolic or enzymatic blockade. By preserving physiological lactylation in nononcogenic contexts, these precision tools may enable the selective dismantling of lactylation‐driven resistance networks while sparing protective immune and metabolic functions.

### 5.5 Combination Strategies and Delivery Platforms

Current evidence favors combination‐based and platform‐enabled development. Lactylation modulation functions as a sensitizing strategy rather than a stand‐alone treatment, reflecting the fact that lactylation‐driven resistance typically operates through multilayered networks that cannot be fully dismantled by monotherapy. Two translational advances—rational combination regimens and advanced delivery platforms—are therefore critical for maximizing therapeutic index while minimizing systemic toxicity.

#### 5.5.1 Combination With Standard Therapies

Lactylation‐targeted agents demonstrate robust synergy with chemotherapy, radiotherapy, and immunotherapy across multiple tumor types, validating their role as universal sensitizers. Stiripentol plus temozolomide overcomes chemoresistance in glioblastoma [231]. C646‐based reversal of T‐cell exhaustion combined with anti‐PD‐1 therapy improves immunotherapy efficacy in gastric cardia adenocarcinoma [216]. Kukoamine A nanotherapy combined with PD‐1 blockade enhances antitumor immunity in HCC [329]. LDHA inhibitors combined with PD‐1 blockade show synergistic antitumor effects in NSCLC and melanoma models [330, 331]. Collectively, these combinations demonstrate a unifying principle: lactylation‐targeted interventions dismantle specific resistance mechanisms—DNA repair proficiency, immune checkpoint stabilization, or metabolic reprogramming—thereby restoring sensitivity to chemotherapy, radiotherapy, or immunotherapy in a mechanism‐guided manner.

#### 5.5.2 Advanced Delivery Platforms

Beyond combination regimens, advanced delivery platforms address two critical challenges: tumor selectivity and dose‐limiting toxicity. By concentrating protein lactylation inhibitors within the TME or restricting their activation to cancer cells, these platforms enable higher effective doses with reduced systemic exposure. TME‐responsive carriers exploit the acidic, lactate‐rich TME to achieve selective drug release. Lactate‐ or pH‐responsive hydrogels and nanoparticles can selectively release protein lactylation inhibitors in the acidic, lactate‐rich TME, minimizing systemic toxicity [332, 333, 334].

Nanoparticle‐based formulations enhance bioavailability, tumor penetration, and controlled release [335, 336, 337, 338]. PLGA–PEG nanoparticle‐based drug delivery systems that effectively target lactylation‐related proteins have been developed, achieving tumor‐targeted, controlled‐release, and biocompatibility [189, 339]. Liposomes carrying siRNAs targeting lactylation‐related genes (e.g., IGF2BP3) improve treatment sensitivity in HCC [188]. These platforms enable genetic or pharmacological disruption of protein lactylation machinery with improved tumor selectivity and reduced off‐target effects.

Engineered biological carriers provide an orthogonal approach. Engineered bacterial vesicles (e.g., ENHL or Lac‐EcN) were employed to deliver lactate, which remodels the tumor immune microenvironment and reverses radiotherapy/immunotherapy resistance [340, 341]. While paradoxical—delivering lactate to overcome resistance—this strategy exploits lactate's dual role: at physiological concentrations, lactate can activate antitumor immunity, while excessive lactate drives immunosuppression. This context‐dependent effect underscores the need for precision dosing and TME‐targeted delivery.

Together, these platform technologies represent a critical translational bridge, enabling lactylation‐targeted agents to achieve therapeutic concentrations within tumors while sparing normal tissues—a prerequisite for safe and effective clinical translation.

#### 5.5.3 Preclinical and Clinical Development Status

The preclinical and clinical development of lactylation‐related therapeutic strategies is still at an early but rapidly evolving stage. Consistent with the rationale discussed above, most current approaches are being advanced as sensitizing or combination strategies rather than stand‐alone therapies, aiming to enhance chemotherapy, radiotherapy, or immunotherapy by disrupting lactate–lactylation‐driven resistance networks. These strategies span multiple intervention levels, including inhibition of lactate production, blockade of lactate transport, modulation of protein lactylation writers or erasers, substrate‐specific targeting, and platform‐enabled delivery.

As shown in Table 1, most lactylation‐targeted interventions remain in preclinical or Phase I/II evaluation, particularly those involving site‐specific lactylation blockade, nanocarrier‐based delivery systems, and engineered biological platforms. Only a limited number of lactylation‐related agents, such as metabolic or lactate transport inhibitors, have entered early clinical evaluation or are being explored through drug repurposing. Future clinical development will require well‐designed trials incorporating pharmacodynamic biomarkers of target engagement, biomarker‐guided patient selection, careful monitoring of metabolic and immune‐related toxicities, and mechanism‐based combinations with standard‐of‐care therapies.

**TABLE 1:** Representative preclinical and clinical development of lactylation‐related therapeutic strategies in cancer.

Intervention level	Representative agent/platform	Target/mechanism	Cancer type	Preliminary findings	Clinical status	Clinical trial number
Lactate production blockade	Stiripentol	LDHA/B; H3K9la–LUC7L2–MLH1 axis	Glioblastoma; HER2‐negative unresectable advanced gastric cancer	Reduces H3K9la and restores TMZ sensitivity in GBM [39]	Drug repurposing/preclinical in oncology	ChiCTR2600121591
Lactate production blockade	D34‐919	ALDH1A3–PKM2; glycolytic flux	Glioblastoma	Suppresses glycolysis and sensitizes GBM to chemoradiotherapy [150]	Preclinical	/
Lactate transport blockade	AZD3965	MCT1	Advanced solid tumors, lymphomas	Enhances radiosensitivity and perturbs DNA damage adaptation [132]	Early clinical evaluation	NCT01791595
Lactate transport blockade	Syrosingopine	Lactate transport; DNA repair‐linked lactylation	Lung adenocarcinoma model	Alters nonhistone lactylation and improves radiotherapy response [342, 343, 344]	Preclinical	/
Lactylation writer targeting	C646	p300/CBP; H3K18la; PDCD1 transcription	Breast cancer, prostate cancer, liver cancer models	Reverses CD8^+^ T‐cell exhaustion and enhances anti‐PD‐1 response [115, 345]	Preclinical/tool compound	/
Lactylation writer targeting	AARS1/AARS2 axis	Global lysine lactyltransferase activity	Bladder cancer, esophageal squamous cell carcinoma	AARS1/2 act as lactate sensors and writers; regulate cGAS lactylation [324, 346]	Preclinical	/
Writer–substrate axis targeting	Kukoamine A nanotherapy	AARS2–AP‐2γ K444la axis	Liver cancer	Disrupts AARS2–AP‐2γ and synergizes with PD‐1 blockade in HCC [329]	Preclinical	/
Writer–lactate interaction blockade	β‐Alanine	AARS‐family lactate binding	Pan‐cancer/tumor development	Interferes with AARS‐dependent lactylation signaling [17]	Preclinical	/
Delactylase modulation	HDAC/SIRT‐related approaches	Histone/nonhistone delactylation	Pan‐cancer	Context‐dependent effects on DNA repair, metastasis, and therapy response [240, 326, 327]	Preclinical/indirect clinical relevance	/
Substrate‐level intervention	MRE11/NBS1‐targeted strategies	DNA repair protein lactylation	Peritoneal metastatic carcinoma refractory to conventional treatment	Impairs homologous recombination and sensitizes tumors [13, 39]	Preclinical/transform clinical exploration	ChiCTR2400083649
Substrate‐level intervention	Irinotecan	BLM‐K24 lactylation	Nonmuscle‐invasive bladder urothelial carcinoma	Suppresses BLM lactylation and reduces chemoresistance [118]	Clinically used drug; exploratory in this context	NCT06766266
Substrate‐level immune regulation	PD‐L1 K280la regulatory axis	AARS1–PD‐L1–HUWE1 axis	Non‐small cell lung cancer	Stabilizes PD‐L1 and promotes immune evasion [51]	Preclinical/biomarker stage	/
Platform‐enabled intervention	Nanocarriers, LOx‐based systems	Tumor enrichment; lactate depletion; immune remodeling	Breast cancer, melanoma	Improve delivery and reshape lactate‐rich TME [337, 347]	Preclinical	/
Platform‐enabled intervention	PROTACs/targeted degraders	HK2, CBP/p300, other upstream regulators	Pancreatic cancer model	Degrade regulators linked to glycolysis and lactylation‐associated programs [348]	Preclinical	/

*Note*: This table includes both direct lactylation‐targeting strategies and lactylation‐related upstream interventions, such as lactate production or transport blockade. For several agents, the lactylation‐related mechanism has been established primarily in preclinical models, whereas clinical evaluation may involve broader metabolic or therapeutic indications.

Abbreviations: ChiCTR, Chinese Clinical Trial Registry; GBM, glioblastoma; HER2, human epidermal growth factor receptor 2; LOx, lactate oxidase; NCT, ClinicalTrials.gov identifier; PROTACs, proteolysis‐targeting chimeras; TMZ, temozolomide.

## 6 Discussion and Outlook

Protein lactylation has added a new layer to our understanding of how tumors convert metabolic stress into durable biological responses. The evidence reviewed here supports three main conclusions: protein lactylation is not merely a passive readout of lactate accumulation, but a regulated posttranslational modification controlled by writers, erasers, and readers; in multiple treatment settings, the lactate–lactylation axis is linked to DNA repair, transcriptional plasticity, immune evasion, and treatment resistance; therapeutic exploration has begun to move from broad lactate suppression toward writer‐related, substrate‐oriented, and delivery‐enabled strategies. Representative protein lactylation events, their modified substrates or sites, proposed mechanisms, phenotypic consequences, therapeutic relevance, and current evidence levels are summarized in Table 2. Most current interventions, however, still act on the wider lactate metabolic network rather than on defined protein lactylation events. This section examines the fundamental research challenges and clinical translation barriers that must be addressed, and outlines future directions for mechanistic investigation and therapeutic development.

**TABLE 2:** Representative lactylation events in cancer organized by functional output and therapeutic relevance.

Functional categories	Substrate/lactylation site	Cancer type/model	Molecular mechanism	Therapy relevance	Evidence level	References
DNA repair (homologous recombination)	NBS1 K388la	Gastric cancer, cervical carcinoma, osteosarcoma, lung cancer, colorectal cancer	Facilitates NBS1 recruitment to DNA break sites, enhances MRN complex assembly, improves HR efficiency	Cisplatin resistance; resensitization by LDHA inhibition (stiripentol); synergy with PARP inhibitors in HR‐deficient contexts	Site‐specific functional validation	[39]
DNA repair (homologous recombination)	MRE11 K673la	Osteosarcoma, breast cancer, colorectal cancer	Enhances DNA binding affinity and end resection efficiency by stabilizing MRE11–DNA interaction	Platinum‐based chemotherapy resistance; potential synthetic lethality with PARP inhibitors	Site‐specific functional validation	[13]
DNA repair (homologous recombination)	RAD51 K73la	Selected resistant contexts	Supports repair capacity in resistant cells	Radiotherapy and chemotherapy resistance	Phenotypic association	[119]
DNA repair (NHEJ)	XLF K288la	Radiation‐exposed models	Stronger interaction with Ku80, more efficient recruitment to radiation‐induced break sites	Radioresistance	Phenotypic association	[152]
DNA repair (homologous recombination)	BLM K24la	Nonmuscle invasive bladder cancer	AARS1‐mediated BLM K24 lactylation stabilizes BLM by blocking MIB1‐dependent ubiquitination, thereby enhancing DNA end resection and homologous recombination repair to drive anthracycline resistance.	Chemotherapy sensitization	Site‐specific functional validation	[118]
DNA repair (homologous recombination)	RPA1 K88la	Lung adenocarcinoma	HAT1‐mediated RPA1 K88 lactylation enhances the binding of RPA1 to ssDNA and HR repair factors, thereby promoting DNA end resection and homologous recombination repair to drive radioresistance in glioblastoma.	Radioresistance	Site‐specific functional validation	[16]
DNA repair (base excision repair/single‐strand break repair)	XRCC1 K247la	Glioblastoma	ALDH1A3‐driven glycolytic reprogramming promotes lactate accumulation and XRCC1 K247 lactylation in glioblastoma stem cells, enhancing XRCC1 nuclear translocation and DNA repair capacity to confer resistance to chemoradiotherapy.	Chemotherapy resistance	Site‐specific functional validation	[150]
Transcriptional reprogramming	H3K14la	Hepatocellular carcinoma	Promotes NEDD4 transcription, accelerates PTEN ubiquitination and degradation, forms reinforcing glycolytic loop	Oxaliplatin and 5‐fluorouracil resistance	Site‐specific functional validation	[117]
Transcriptional reprogramming	H3K18la at IGF2BP3 promoter	Colorectal cancer	Promotes IGF2BP3 transcription, stabilizes HDGF mRNA in m^6^A‐dependent manner	Oxaliplatin resistance	Locus‐level evidence	[349, 350]
Transcriptional reprogramming	H3K18la at MFGE8 promoter	Cancer‐associated fibroblasts (CAFs)	Promotes MFGE8 secretion, supports tumor cell survival	Chemotherapy resistance (stromal mechanism)	Locus‐level evidence	[89, 351]
Transcriptional reprogramming	H3K18la at CCNB2 promoter	Pancreatic cancer	CAF‐derived lactate induces H3K18la, upregulates CCNB2, and drives glycolytic reprogramming.	Chemotherapy resistance (tumor–stroma feedback)	Locus‐level evidence	[352]
Transcriptional reprogramming	H3K18la at CD274 (PD‐L1) promoter	Hepatocellular, gastric, colorectal cancers	c‐MYC‐mediated transcriptional upregulation of PD‐L1	Immune checkpoint blockade resistance	Locus‐level evidence	[59, 94, 208]
Transcriptional reprogramming	H3K18la at YTHDF2 promoter	Pancreatic cancer, colorectal cancer	p300 mediates H3K18la enrichment at the IGF2BP2 promoter, enhances IGF2BP2 transcription, and is involved in m^6^A‐related posttranscriptional regulation, tumor progression, and immune microenvironment remodeling.	Immune escape/resistance to immunotherapy	Locus‐level evidence	[353, 354]
Transcriptional reprogramming	H3K18la at PD‐L1 promoter	Ovarian cancer	H3K18la is enriched at the PD‐L1 promoter, boosts PD‐L1 transcription, and promotes immune evasion.	Immune escape	Locus‐level evidence	[355]
Transcriptional reprogramming	H3K18la at ARG1 promoter	Tumor‐associated macrophages	Promotes ARG1 transcription, depletes arginine, impairs T‐cell function	Chemo‐immunotherapy resistance	Locus‐level evidence	[6, 137, 295, 356]
Transcriptional reprogramming	H3K18la at RUBCNL/pacer locus	Colorectal cancer	EP300‐catalyzed H3K18la upregulates RUBCNL/pacer, enhances autophagosome maturation	Bevacizumab (anti‐VEGF) resistance	Site‐specific functional validation	[131]
Transcriptional reprogramming	H3K18la at HECTD2 promoter	Hepatocellular carcinoma	Promotes HECTD2 transcription, creates lactate–lactylation–antioxidant reinforcing loop	Lenvatinib resistance	Locus‐level evidence	[189]
Transcriptional reprogramming	H3K18la at BMAL1 locus	Glioblastoma	Promotes BMAL1 transcription and nuclear translocation, enhances VEGFA signaling	Bevacizumab resistance	Locus‐level evidence	[194]
Transcriptional reprogramming	H3K18la at B7‐H3 promoter	Glioblastoma	Lactic acid induces H3K18la to directly bind to the B7‐H3 promoter, promoting B7‐H3 expression and immune evasion.	Immune escape	Locus‐level evidence	[357]
Transcriptional reprogramming	H3K18la at NFS1 promoter	Liver cancer	H3K18la is enriched at the NFS1 promoter, boosts NFS1 transcription, lowers sensitivity to ferroptosis, and promotes posttreatment metastasis.	Oxaliplatin resistance	Locus‐level evidence	[241]
Transcriptional reprogramming	H3K18la at ACAT2 promoter	Stomach cancer	H3K18la mediates VCAM1 transcription activation, which then promotes invasion, migration, and an immunosuppressive environment through signals like AKT/mTOR/CXCL1.	Resistance to immunotherapy	Locus‐level evidence	[358]
Transcriptional reprogramming	H3K18la at VCAM1 promoter	Pancreatic cancer	H3K18la is specifically enriched at the ACAT2 promoter and activates ACAT2 transcription, boosting cholesterol metabolism and sEV–cholesterol‐mediated immune suppression.	Immune suppression	Locus‐level evidence	[288]
Transcriptional reprogramming	H3K18la at PDCD1 promoter	Gastric cardia adenocarcinoma	H3K18la is enriched at the PDCD1 promoter, promoting PD‐1 expression.	resistance to immunotherapy	Locus‐level evidence	[216]
Transcriptional reprogramming	H3K18la at PDIA6 promoter	Gastric cancer dendritic cells	ER stress induces H4K12la, promotes PDIA6 expression, attenuates dendritic cell immune activity.	Anti‐PD‐1 resistance	Locus‐level evidence	[215]
Transcriptional reprogramming	H3K9la–IL‐11 axis	Head and neck squamous cell carcinoma	H3K9la promotes IL‐11 expression, activates JAK2/STAT3, and weakens CD8^+^ T‐cell activity.	Immunotherapy resistance	Phenotypic association	[222]
Transcriptional reprogramming	H3K9la at LUC7L2 promoter	Glioblastoma	H3K9la activates LUC7L2 transcription, causing MLH1 intron retention and weakening mismatch repair.	Temozolomide resistance	Locus‐level evidence	[231]
Transcriptional reprogramming	H4K12la at SLFN5 promoter	Triple‐negative breast cancer	Histone lactylation‐associated regulation of SLFN5 expression	Indirect relevance to treatment tolerance	Locus‐level evidence	[98]
Transcriptional reprogramming	H4K12la at RAD23A promoter	Ovarian cancer	H4K12la activates super‐enhancer‐driven RAD23A expression, boosting DNA damage repair capacity.	PARP inhibitor resistance	Locus‐level evidence	[226]
Transcriptional reprogramming	H4K12la at GCLC promoter	Colorectal cancer	H4K12la activates GCLC transcription, boosts GSH production, and inhibits ferroptosis.	Chemotherapy resistance	Locus‐level evidence	[31]
Transcriptional reprogramming	H3K18la (chromatin accessibility)	Irradiated tumor models	Enhances chromatin accessibility, facilitates repair factor access to damaged DNA	Radioresistance	Phenotypic association	[90]
Immune checkpoint regulation	PD‐L1 K280la	Various tumor types	Enhances PD‐L1 protein stability by mitigating ubiquitin‐dependent degradation	Immune checkpoint blockade resistance	Site‐specific functional validation	[51]
Immune checkpoint regulation	PD‐L1 K189 delactylation	Various tumor types	Facilitates nuclear translocation, augments tumor‐promoting transcriptional activity	Immune checkpoint blockade resistance	Site‐specific functional validation	[209]
Immunosuppression (Treg)	MOESIN lactylation	Regulatory T cells	Enhances Treg stability and function	Anti‐PD‐1 resistance	Phenotypic association	[217]
Immunosuppression (Treg)	APOC2 K70la	Non‐small cell lung cancer, Tregs	Upregulates immunosuppressive pathways in Tregs, promotes suppressive function, limits antitumor immunity	Anti‐PD‐1 resistance, metastasis	Site‐specific functional validation	[218]
Myeloid immunosuppression	METTL3 lactylation	Tumor‐infiltrating myeloid cells	Enhances Jak1 translation, amplifies JAK1/STAT3 signaling, suppresses proinflammatory activity	Immunotherapy resistance	Site‐specific functional validation	[58]
Metabolic reprogramming	IGF2BP3 K76la	Hepatocellular carcinoma	Promotes m^6^A‐related regulation of PCK2 and NRF2 transcripts, reprograms serine metabolism, strengthens antioxidant defense	Lenvatinib resistance	Site‐specific functional validation	[188]
Antioxidant defense	HECTD2 lactylation	Hepatocellular carcinoma	Promotes KEAP1 degradation, activates KEAP1/NRF2 pathway	Lenvatinib resistance	Site‐specific functional validation	[189]
Protein stabilization	DCBLD1 K172la	Cervical cancer	HIF‐1α‐mediated DCBLD1 lactylation stabilizes protein, inhibits autophagic degradation of G6PD, and activates PPP.	Proliferation and metastasis (relevant to targeted therapy bypass)	Site‐specific functional validation	[112]
Protein stabilization	TFEB K91la	Pancreatic cancer	Blocks TFEB interaction with WWP2, reduces ubiquitination and proteasomal degradation, enhancing autophagy–lysosome activity	Autophagy‐associated therapy tolerance	Site‐specific functional validation	[33]
Protein stabilization	AKR1B10 K173la	HCC	AARS1‐mediated lactylation stabilizes AKR1B10 and supports AKR1B10–LDHA–H3K18la glycolytic feedback.	Lenvatinib resistance	Site‐specific functional validation	[359]
Protein stabilization	PTBP1 K436la	Glioma stem cells	Inhibits TRIM21‐mediated proteasomal degradation and enhances PTBP1 RNA‐binding capacity, stabilizing PFKFB4 mRNA	GBM stemness and glycolytic resistance vulnerability	Site‐specific functional validation	[303]
Stress survival signaling	VPS34 K356/K781la	Selected tumor models (radiotherapy contexts)	Autophagy‐mediated stress survival; radioresistance	Radiotherapy resistance; targetable by VPS34 inhibitors (e.g., SAR405)	Site‐specific functional validation	[164, 360, 361]
Tumor suppressor inhibition	p53 K120/K139la	Multiple tumor models with wild‐type p53	AARS1‐mediated p53 lactylation impairs p53 liquid–liquid phase separation and DNA binding, suppressing p53 transcriptional activity.	Restoring p53 tumor suppressor activity; β‐alanine/AARS1 axis	Site‐specific functional validation	[17]
Metabolic bypass	PKM2 lactylation	Colorectal cancer	Promotes vasculogenic mimicry and bevacizumab resistance by facilitating FOSL1 super‐enhancer formation	Bevacizumab resistance	Site‐specific functional validation	[361]
Metabolic bypass	ENO1 K71la K89la	Osimertinib‐resistant lung adenocarcinoma, ovarian cancer	ENO1 lactylation links metabolic restoration to persistent signaling in EGFR‐TKI resistance; in ovarian cancer, drives metabolic reprogramming and angiogenesis under bevacizumab pressure	EGFR‐TKI resistance (osimertinib); bevacizumab resistance	Phenotypic association	[184, 193]
Driving metastasis and angiogenesis	PKM2 K206la	Colorectal cancer	PKM2 lactylation links lactate‐driven metabolic adaptation to persistent transcriptional signaling in bevacizumab resistance; in colorectal cancer, it promotes PKM2 nuclear translocation, facilitates FOSL1 super‐enhancer formation, and drives a vasculogenic mimicry program.	Bevacizumab resistance	Site‐specific functional validation	[195]
Metabolic reprogramming	PFKP K688/K392la	Selected tumor models	PFKP lactylation connects metabolic stress adaptation with persistent glycolytic reprogramming; in ovarian cancer, it enhances PFKP activity to sustain ATP production and lactate generation.	Chemotherapy and targeted therapy resistance	Moderate evidence	[362, 363]
Metabolic bypass	G6PD K45la	Selected tumor models	G6PD lactylation connects metabolic reprogramming with oxidative stress tolerance; by activating the pentose phosphate pathway and increasing NADPH production, it reinforces antioxidant defense and supports tumor cell survival under therapeutic pressure.	Radiotherapy and chemotherapy resistance	Moderate evidence	[156, 246, 247]
Metabolic bypass	DCBLD1 K172la	Cervical cancer	DCBLD1 K172 lactylation links lactate‐driven protein stabilization to redox metabolic reprogramming; by stabilizing DCBLD1 and inhibiting autophagic degradation of G6PD, it activates the pentose phosphate pathway and increases NADPH production.	Proliferation and metastasis; relevant to targeted therapy bypass	Site‐specific functional validation	[112]
Cancer stemness	SHMT2 lactylation (site not fully mapped)	Selected tumor models	SHMT2 lactylation links metabolic adaptation to one‐carbon pathway rewiring; it modulates SHMT2 activity, reshapes serine–glycine–one‐carbon metabolism, and supports nucleotide synthesis and methylation reactions required for tumor cell survival.	Chemotherapy resistance	Emerging evidence	[304]
Metabolic bypass	NUSAP1 K34la	Selected tumor models	NUSAP1 lactylation establishes a feed‐forward connection between protein stabilization and glycolytic signaling; by reinforcing the NUSAP1–LDHA feedback loop, it sustains lactate production and amplifies lactylation‐dependent signaling.	Drug tolerance and resistance	Emerging evidence	[364]
Therapy resistance	EGR1–lactate feedback loop	EGFR‐TKI‐resistant models	NNMT connects metabolic reprogramming with EGR1‐dependent transcriptional signaling; it promotes EGR1 expression, increases lactate production, and establishes a lactate–EGR1 feed‐forward circuit that sustains therapeutic resistance.	EGFR‐TKI resistance	Phenotypic association	[187]
Therapy resistance	IGF2BP3 K76la → PCK2/NRF2	Hepatocellular carcinoma	IGF2BP3 K76 lactylation links lactate‐driven m^6^A regulation to serine metabolic reprogramming; by stabilizing PCK2 and NRF2 mRNAs, it increases SAM availability for m^6^A modification and strengthens antioxidant defense.	Lenvatinib resistance	Site‐specific functional validation	[188]
Therapy resistance	Metabolic enzymes (unspecified)	Irradiated tumor models	Lactylation affects NADPH‐generating flux, antioxidant defense, and stress‐buffering programs.	Radiotherapy resistance	Phenotypic association	[156]

Abbreviations: AKR1B10, aldo‐keto reductase family 1 member B10; B7‐H3, B7 homolog 3; CCNB2, cyclin B2; HDGF, hepatoma‐derived growth factor; IGF2BP2, insulin‐like growth factor 2 mRNA‐binding protein 2; MFGE8, milk fat globule‐EGF factor 8; MIB1, mindbomb E3 ubiquitin protein ligase 1; NUSAP1, nucleolar and spindle‐associated protein 1; PFKP, phosphofructokinase, platelet type; SAM, S‐adenosylmethionine; sEV, small extracellular vesicle; ssDNA, single‐stranded DNA.

### 6.1 Fundamental Research Challenges

Several unresolved issues continue to limit mechanistic interpretation and translational potential.

#### 6.1.1 Detection Technologies With Spatial Resolution

Many studies still depend on pan‐Kla antibodies, candidate‐site validation, or bulk proteomics—approaches that differ substantially in specificity, sensitivity, and spatial resolution. Antibody‐based assays may crossreact with structurally related lysine acylations, whereas mass spectrometry requires stringent site assignment and adequate sample quality [365, 366, 367]. Bulk tumor profiling also masks cell‐specific protein lactylation patterns, making it difficult to determine which cell type drives the observed phenotype [368, 369, 370, 371]. Achieving molecular precision with spatial context represents a critical technical barrier for clinical translation. Emerging technologies—spatial proteomics, single‐cell multiomics, and chemoproteomic probes—offer promise for resolving cell‐type‐specific protein lactylation programs [371], though require further optimization. Integrating spatial transcriptomics with protein lactylation profiling could reveal how tumor, immune, and stromal compartments differentially regulate protein lactylation networks—essential for compartment‐specific therapeutic strategies.

#### 6.1.2 Functional Validation Beyond Correlative Observations

Increased Kla is often observed in aggressive tumors or resistant models, but this does not prove that a given protein lactylation site drives the phenotype. Some changes may simply reflect high glycolytic activity, lactate accumulation, or treatment‐induced metabolic stress—a concern particularly relevant when interpreting LDH, MCT, or glycolysis‐targeting interventions, which can alter pH, redox balance, ATP production, and lactate‐dependent signaling in addition to protein lactylation. Distinguishing lactate‐mediated effects from lactylation‐specific effects represents a fundamental challenge. Rigorous functional validation requires site‐directed mutagenesis, genetic code expansion, and confirmation in vivo. In addition, distinguishing K_L‐la_ from K_D‐la_ will require stereoisomer‐resolved mass spectrometry or rigorously validated stereospecific reagents combined with isotope tracing. These approaches are particularly important in treatment–response studies, in which changes in total Kla may reflect several metabolically distinct pathways [13, 32, 372]. Without this level of rigor, distinguishing driver protein lactylation events from passenger modifications remains difficult. High‐throughput genetic code expansion platforms combined with CRISPR screens may enable systematic interrogation of protein lactylation sites, accelerating driver event identification.

#### 6.1.3 Context‐Dependent Regulation Across Tumor Ecosystems

The biological consequences of protein lactylation are context dependent. Different cancer types vary in lactate production, oxygenation, stromal composition, and reliance on specific transporters or writers. Even within the same tumor, a protein lactylation event may have distinct effects in malignant cells versus immune or stromal cells [368, 369]. Rather than a single “lactylation signature,” the field must embrace cell‐type‐specific, microenvironment‐contextualized understanding. The regulatory landscape also remains incomplete. p300/CBP, HDACs, and SIRTs regulate several lysine acylations, complicating attribution of drug effects to protein lactylation alone. The identification of AARS1/2 as lactate‐sensing lactyltransferases has provided a stronger enzymatic basis [18], but nonenzymatic mechanisms may also contribute when lactyl‐CoA levels are high [70]. Recently identified lactylation readers—Brg1, DPF2, and TRIM33—represent only the tip of the iceberg; comprehensive reader identification remains essential.

Protein lactylation coexists with acetylation, crotonylation, succinylation, ubiquitination, and phosphorylation, yet how these modifications compete or cooperate at shared residues remains poorly defined [6, 373, 374, 375]. PTM crosstalk likely operates through multiple mechanisms: direct competition, hierarchical modification cascades, and combinatorial codes. Understanding these interactions will require systematic mapping using multi‐PTM proteomics and functional validation of combinatorial modification states.

### 6.2 Clinical Translation Barriers

Beyond fundamental research challenges, clinical translation faces several practical barriers.

#### 6.2.1 Therapeutic Specificity and Safety Concerns

Lactylation‐modifying enzymes often exhibit pleiotropic functions, regulating multiple lysine acylations and raising concerns about off‐target toxicity [6, 376, 377, 378]. Site‐specific interventions offer higher specificity but may prove insufficient when multiple protein lactylation events contribute to resistance. Peptide‐based inhibitors often suffer from poor cell permeability, short half‐life, and limited tumor penetration [379]. Protein lactylation also participates in physiological processes—inflammatory resolution, stem cell pluripotency, and osteoblast differentiation [75, 380]—meaning nonselective inhibition may interfere with normal functions. Tumor‐selective delivery strategies and biomarker‐guided patient selection will be essential to minimize these risks.

#### 6.2.2 Biomarker Development and Metabolic Heterogeneity

Effective clinical translation requires biomarkers that can identify patients most likely to benefit from lactylation‐targeted therapies. Candidate biomarkers include tumor lactate burden, MCT1/MCT4 expression, AARS1/2 status, site‐specific Kla readouts, and immune contexture [18, 381, 382, 383, 384]. These biomarkers should be used not only for patient selection but also for pharmacodynamic monitoring to confirm target engagement. Most protein lactylation biomarkers, however, remain in the discovery stage and require validation in large prospective cohorts using standardized detection methods [100, 358, 385, 386, 387]. Metabolic heterogeneity across tumor types, clinical stages, and cell populations further complicates therapeutic effectiveness [388, 389, 390, 391]. Tumor‐type‐specific differences in MCT dependency, SIRT function, and lactylation‐driven resistance mechanisms necessitate personalized intervention strategies. Combination therapies targeting multiple nodes in the lactate–lactylation axis may be required to overcome compensatory metabolic pathways.

#### 6.2.3 Clinical Trials

Clinical translation requires well‐designed trials that incorporate pharmacodynamic biomarkers to confirm target engagement, enrich enrollment for patients with high lactylation signatures, pair protein lactylation inhibitors with standard therapies based on mechanistic rationale, monitor for metabolic and immune‐related adverse events, and consider adaptive trial designs for evaluating therapies across multiple tumor types [289, 386, 392, 393]. Currently, only a few lactylation‐related agents have entered clinical trials, with AZD3965 (MCT1 inhibitor) completing Phase I/II trials demonstrating pharmacokinetic profile. No lactylation‐selective therapy has yet shown validated efficacy in patients. Near‐term progress is therefore more likely to come from biomarker‐guided combinations than from stand‐alone protein lactylation inhibitors.

### 6.3 Future Directions

The next stage of the field should move beyond reporting global Kla changes and focus on identifying which lactylation sites are reproducible, functional, and therapeutically actionable. This requires site‐level Kla detection, functional perturbation of modified residues, rescue of downstream activity, and confirmation in disease‐relevant models. The complete enzymatic machinery governing protein lactylation remains incompletely characterized, warranting systematic identification through CRISPR screening, structural biology, and chemoproteomic profiling. Understanding crosstalk with other posttranslational modifications will require advanced proteomic technologies to map modification hierarchies and combinatorial codes. Clinical translation remains early, with near‐term progress more likely to come from biomarker‐guided combinations using tumor lactate burden, MCT expression, AARS status, immune contexture, and validated site‐specific Kla readouts for patient selection and pharmacodynamic monitoring.

### 6.4 Concluding Remarks

Protein lactylation provides a useful framework for linking tumor metabolism with epigenetic regulation, protein function, immune suppression, and treatment resistance. The current evidence supports its biological relevance but not yet its status as a clinically established target class. The field now needs more rigorous detection technologies with site‐level specificity and spatial resolution, stronger site‐level causality through functional validation, clearer separation between lactate effects and protein lactylation effects, better definition of cancer‐ and cell‐type‐specific dependencies, and biomarker‐guided clinical trials pairing lactylation‐targeted interventions with standard therapies. Until these gaps are addressed, protein lactylation should be viewed as a promising but still maturing therapeutic axis, with the most realistic near‐term value in mechanism‐guided biomarkers and rational combination strategies. As the field progresses from descriptive observations to mechanistic validation and clinical translation, lactylation‐targeted therapies may provide new opportunities to overcome treatment resistance and improve patient outcomes.

## Author Contributions

W.T.H., P.H., S.W., and X.D.Z. conceived the structures and concept of this review and drafted the first version. Y.Y.C. designed, prepared, and revised most of the figures and tables. Y.Y.Y. contributed to the conceptualization and revision of the manuscript. S.W. and X.D.Z. helped with the first version of some figures. P.H. contributed to the literature searching and table preparation. Z.R.Y. and S.Q.C. helped with grammar and spelling checking. Y.Y.Y. and F.G. provided guidance and financial support. W.T.H., P.H., S.W., and X.D.Z. contributed equally to the first author. All the authors have read and approved the article.

## Funding

This work was supported by the National Natural Science Foundation of China (No. 82273579; No. 82473580; No. 82574032; No. 82674606) and the Academic General project (No. 2023MS006).

## Conflicts of Interest

The authors declare no conflicts of interest.

## Ethics Statement

The authors have nothing to report.

## Data Availability

The authors have nothing to report.
